# Improved modeling of human AD with an automated culturing platform for iPSC neurons, astrocytes and microglia

**DOI:** 10.1038/s41467-021-25344-6

**Published:** 2021-09-01

**Authors:** Reina Bassil, Kenneth Shields, Kevin Granger, Ivan Zein, Shirley Ng, Ben Chih

**Affiliations:** 1 Department of Biochemical and Cellular Pharmacology, Genentech Inc., South San Francisco, CA USA; 2 Division of Biological Sciences, Section of Neurobiology, University of California, San Diego, La Jolla, CA USA; 3 Department of Neuroscience, Genentech Inc., South San Francisco, CA USA

**Keywords:** Stem-cell biotechnology, Drug screening, Alzheimer's disease

## Abstract

Advancement in human induced pluripotent stem cell (iPSC) neuron and microglial differentiation protocols allow for disease modeling using physiologically relevant cells. However, iPSC differentiation and culturing protocols have posed challenges to maintaining consistency. Here, we generated an automated, consistent, and long-term culturing platform of human iPSC neurons, astrocytes, and microglia. Using this platform we generated a iPSC AD model using human derived cells, which showed signs of Aβ plaques, dystrophic neurites around plaques, synapse loss, dendrite retraction, axon fragmentation, phospho-Tau induction, and neuronal cell death in one model. We showed that the human iPSC microglia internalized and compacted Aβ to generate and surround the plaques, thereby conferring some neuroprotection. We investigated the mechanism of action of anti-Aβ antibodies protection and found that they protected neurons from these pathologies and were most effective before pTau induction. Taken together, these results suggest that this model can facilitate target discovery and drug development efforts.

## Abstract (web summary)

Human induced pluripotent stem cell (iPSC) cells have been used to model disease in specific cell types. Here, the authors develop an automated long-term culturing platform of human iPSC neurons, astrocytes, and microglia and use it to model some cellular aspects of Alzheimer’s disease.

## Introduction

Human iPSCs have become powerful tools in modeling human diseases and hold tremendous potential for translational research in target discovery and drug development. Human iPSC-derived neurons are sensitive and require extended culturing time (80 days) to develop mature neuron characteristics^1^. Long-term neuronal cell maintenance proves challenging using traditional manual techniques and thus, most small-molecule and CRISPR screens were conducted using neurons cultured for <30 days^2–4^. Given that many neurodegenerative diseases are adult onset, such as Alzheimer’s disease (AD), high-throughput screening platforms combined with longer neuronal culture times could be more translationally relevant. With the development of modern automation technology and the increased use of human iPSC for disease modeling, increased demand and implementation of automated culturing platforms for iPSC neurons are anticipated.

AD is characterized by the pathological hallmarks of amyloid-β (Aβ) plaques, neurofibrillary tangles, astrogliosis, and neuronal loss. Amyloid plaques are composed of aggregated Aβ peptides, often surrounded by Phospho-Tau (pTau) positive dystrophic neurites (neuritic plaques) and activated microglia. Neurofibrillary tangles contain hyperphosphorylated Tau, with increased phosphorylation at several amino acid sites^5–9^. Additional previously identified AD pathologies include cerebrovascular amyloid angiopathy, microgliosis, neuroinflammation, and major synaptic alteration^8,10,11^.

The amyloid hypothesis proposes that abnormally folded Aβ peptides initiate a causal cascade beginning with Aβ oligomer aggregation into plaques, which then trigger Tau hyperphosphorylation and neurofibrillary tangle formation, ultimately resulting in neuronal cell death^12,13^. This hypothesis has been the theoretical foundation for the generation of numerous animal models, diagnostics, and drug development programs for AD^12^. Supporting some aspects of this hypothesis, rodent AD models often overexpress mutated forms of the familial AD (FAD)-causing genes, *APP* and/or *PSEN*, leading to overproduction of Aβ peptides, extensive amyloid plaque formation, neuroinflammation, and some synaptic dysfunction^14,15^. However, important aspects of AD pathology such as p-Tau induction and severe neuronal loss have not been robustly established^10,16,17^. The recent failures of many anti-Aβ therapeutics have led some in the field to cast some doubt on the amyloid hypothesis^18–20^. Therefore, the relevance of existing rodent models for AD drug development is still debated^14,17,21^. Without robust animal, cellular, or translational models, the mechanisms by which Aβ oligomers trigger p-Tau induction and neuronal death have remained elusive; there are currently no approved disease-modifying treatments for AD despite 40 years of intense research efforts.

For this reason, the development of improved model systems that more robustly mimic human AD pathophysiology is important for drug development and translation. Innovations in developing human induced pluripotent stem cell (iPSC) neuronal and microglial differentiation protocols have opened up new possibilities in translational models for human disease^22^. Recent studies show that 3D cultures of human neurons overexpressing mutant APP in vitro led to pTau induction^23^. Furthermore, implanting human iPSC neurons into AD mouse models recapitulates pTau induction and human neuronal sensitivity phenotypes not previously observed in traditional mouse models^24^. While more translationally relevant, the techniques described above can be labor-intensive and highly variable, and thus not ideal for drug screening and development.

Building on previous findings that human neurons could be more translationally relevant to AD pathology, we aimed to generate an experimental human iPSC neuron culturing platform that is quantitative, high throughput, multiplexed, systematic, and reproducible to allow for pharmacological studies, mechanistic studies, and screening efforts. Here we present a high-throughput human iPSC-based model of AD that recapitulates key hallmark pathologies that have been historically difficult to replicate in one model system. This model recapitulates robust Aβ plaque formation with surrounding pTau positive dystrophic neurites and human iPSC microglia in vitro. Consistent with AD pathologies, we observed severe synapse loss, axon degeneration, and pTau induction resulting in severe neuronal loss. Using this model, we demonstrated three utilities: (1) We conducted a focused proof-of-concept compound library screen. We identified known kinase pathways—such as glycogen synthase kinase 3 (GSK3), Fyn, and dual leucine zipper kinase (DLK)—that have previously been implicated in AD, thereby validating the system as a useful screening tool. (2) We explored mechanisms of microglia-driven plaque formation. (3) Finally, we used our model to investigate the mechanism of action of anti-Aβ antibodies and our findings highlight the importance of early administration and high exposure of therapeutic compounds^25–27^. Overall, we have generated a robust platform that could facilitate target discovery, drug development, and impactful MoA studies in AD research toward a potential treatment.

## Results

### Generation of a high-throughput, automated iPSC-derived human neuron culturing platform

**Fig. 1:**
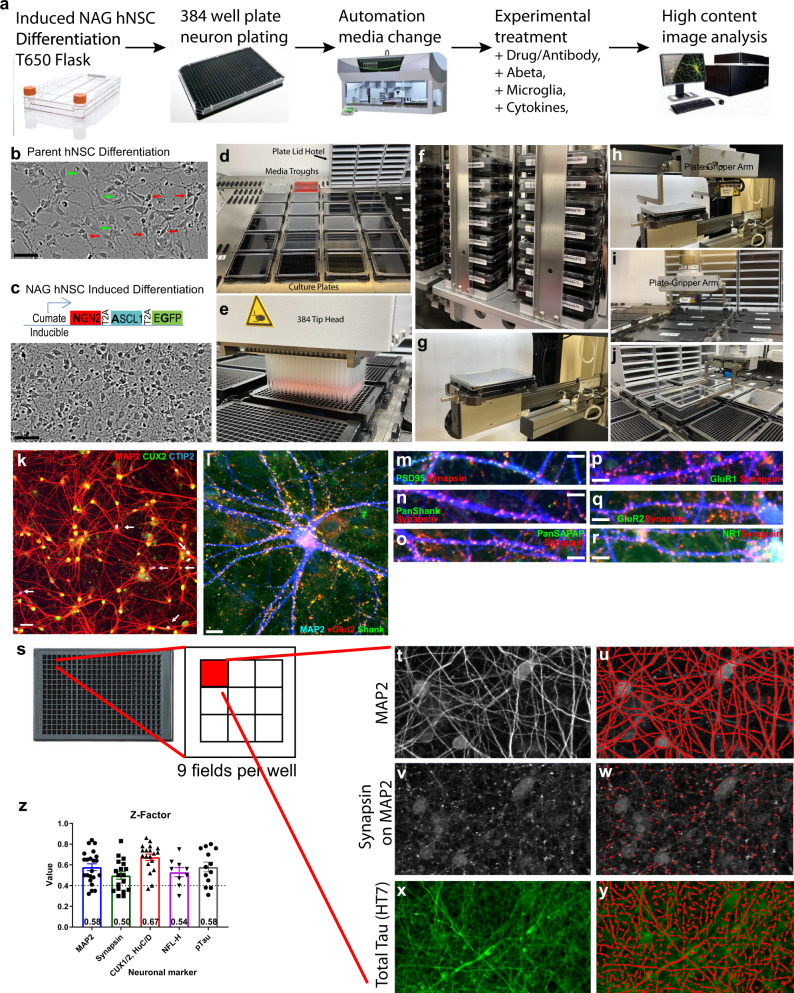
A high-throughput, automated human iPSC-derived neuron differentiation and culturing platform. **a** Schematic workflow of human iPSC neuron differentiation, plating, maintenance, and maturation with automated media change using Fluent liquid handler (Tecan). Mature culture (12 weeks+) is ready for various experimental treatments and conditions. At the end of the experiments, fixed cells are processed for immunostaining using automated plate washers and then quantified with high content image analysis via IN Cell Analyzer 6000 (GE). **b** Representative images of unsynchronized, heterogeneous WT iPSC-derived NSC differentiation (red arrows indicate differentiated neurons; green arrows indicate undifferentiated NSCs). Scale bar 50 μm. **c** Stable expression of cumate-inducible NAG construct and treatment with cell cycle inhibitors synchronizes and homogenizes human iPSC neuron differentiation. Scale bar 50 μm. **d** Representative image of 20 culture plate media change using Fluent workstation (Tecan). **e** Fluent 384 tip liquid handler head consistently and systematically removes old media and adds new media across all wells per plate. **f** Integrated incubator and barcoded plates enable automated plate tracking and care. **g** Automated plate ejection from incubator and **h** gripper arm retrieves plate then **i** places it on plate deck for subsequent media change. **j** Gripper arm removes the lid and places it on plate-lid hotel during media change. **k** Differentiated NAG neurons express dendritic marker MAP2 (red), layer II/III cortical marker CUX2 (green), with a small subpopulation expressing layer V/VI marker CTIP2 (blue) indicated by white arrows. Scale bar = 50 μm. **l**–**r** Mature NAG neurons express dendritic marker MAP2 (blue), synaptic markers VGLUT2 (red), Shank (green), scale bar = 20 μm. **l** Synapsin (red), PSD95 (green), scale bar = 10 μm (**m**), Pan SHANK (green) (**n**), PanSAPAP (green) (**o**), GluR1 (green) (**p**), GluR2 (green) (**q**), and NR1 (green) (**r**). **s** High content image analysis are made from 9 fields/well in a 384-well plate covering 70% well area. **t**–**y** Examples of image analysis using IN Cell Developer toolbox companion software to quantitate phenotypes such as dendrites (**t**, **u**), synapses (**v**, **w**), and axons (**x**, **y**) in an automated, systematic, and unbiased way. Careful scripts were developed to isolate exact regions of interest are shown in red on the right panels. Multiple measurements such as total area, total intensity, and count are made for each marker. **z** Z-factors are calculated from results using a neuron culturing platform and high content image analysis software. Z-factors are in the range from 0.5 to 0.75 and averaged from 10 to 20 different experiments using different batches of neurons. Each experiment with four wells, 1000+ neurons/well quantified. Data are presented as mean values +/− SEM and *n* = 4 wells.

The workflow (Fig. 1a) started with induced iPSC neuron differentiation in large batches (100–200 million cells), which were then replated into 384-well imaging plates. Fluent^®^ automation workstation (Tecan) was used for multiple liquid-handling steps such as cell plating, media changes, experimental treatment, and cell fixation to achieve systematic, reproducible, and precise neuron handling. The multiplex-stained cells were then scanned and quantified using an automated high content imaging system, IN Cell Analyzer 6000 (GE Healthcare).

To achieve accelerated, synchronized, and homogeneous differentiation, we generated two different human iPSC-derived neural stem cells (NSC) lines expressing NGN2, ASCL1, and a green fluorescent protein (GFP) reporter under a cumate-inducible system^28–31^. Cumate induction in combination with cell cycle inhibition (PD0332991) in both NSC lines generated homogeneous iPSC neurons within 7 days as expected (Fig. 1b, c). After neuron differentiation and replating, primary human astrocytes were added to the culture to promote neuronal health and maturation.

The Fluent automation workstation was used to maintain long-term iPSC neuronal cultures in 384-well plates. Convenience features of the automated workstation allowed walk-away implementation to maintain consistent and healthy neurons for up to 6 months (Fig. 1d–j). The resulting neurons from both NSC lines were homogenous, upper-layer cortical neurons with over 95% expressing CUX2 with only 2–5% expressing CTIP2 or SATB2 (Fig. 1k). Neurons also had extensive synaptic connections and expressed several pre- and post-synaptic markers: PSD95, SHANK, PanSHANK, GluR1, GluR2, vGLUT2, Synapsin 1/2, PanSAPAP, and NR1 (Fig. 1l–r). The use of 384-well plates enabled simultaneous testing of numerous experimental conditions, each with four biological replicates (*n* = 4). IN Cell Analyzer 6000 and ImageXpress Micro Confocal and their respective softwares, were used for automated confocal image acquisition and analysis. We imaged nine fields per well, which covered 70% of the well area, and captured over 1000 neurons (Fig. 1s). Image analysis scripts provided precise segmentation of markers of interest including dendrites (MAP2), cell bodies (CUX2), axons (Tau, p-Tau), and synapses (Synapsin 1/2) (Fig. 1t–y). To characterize the variability of the assay performance, average z-factors (a measure of assay reliability) were calculated from multiple batches and experiments (10–20) for the aforementioned assays. They ranged from 0.5 to 0.7 (Fig. 1z) indicating robust assay.

### An in vitro human neuron model of Alzheimer’s disease recapitulates AD pathology hallmark and kinetics

To investigate whether AD pathologies can be studied in a controlled, in vitro system of human neurons, we treated cultured human iPSC-derived neurons with synthetic Aβ42 oligomers, prepared by oligomerization of Aβ42 monomers at 4 °C following previously published protocols (Supplementary Fig. 1A)^32^. To reduce variability between Aβ42 oligomer preps, we optimized the oligomerization duration and selected Aβ42 peptide lots that demonstrated consistent AD pathologies in neurons upon treatment: synapse loss, pTau induction, and map2 area reduction (Supplementary Fig. 1A–J). We also developed Aβ oligomer selective and Aβ fibril selective ELISAs to confirm the generation of oligomer species (Supplementary Fig. 1E–G). We found that our preps likely contain a heterogeneous mixture of both soluble oligomer and fibrils, thus we refer to these preps as “soluble Aβ42 species” (sAβ42s). We found that sAβ42s-induced neurotoxicity is specific to human neurons, as primary rat cortical neurons treated with several different lots of Aβ42 oligomer preparations did not show a reduction in dendrites or synapses (Supplementary Fig. 1N, O). Identified Aβ42 monomer lots were purchased in bulk and 3 similarly performing Aβ42 lots were used throughout this paper.

**Fig. 2:**
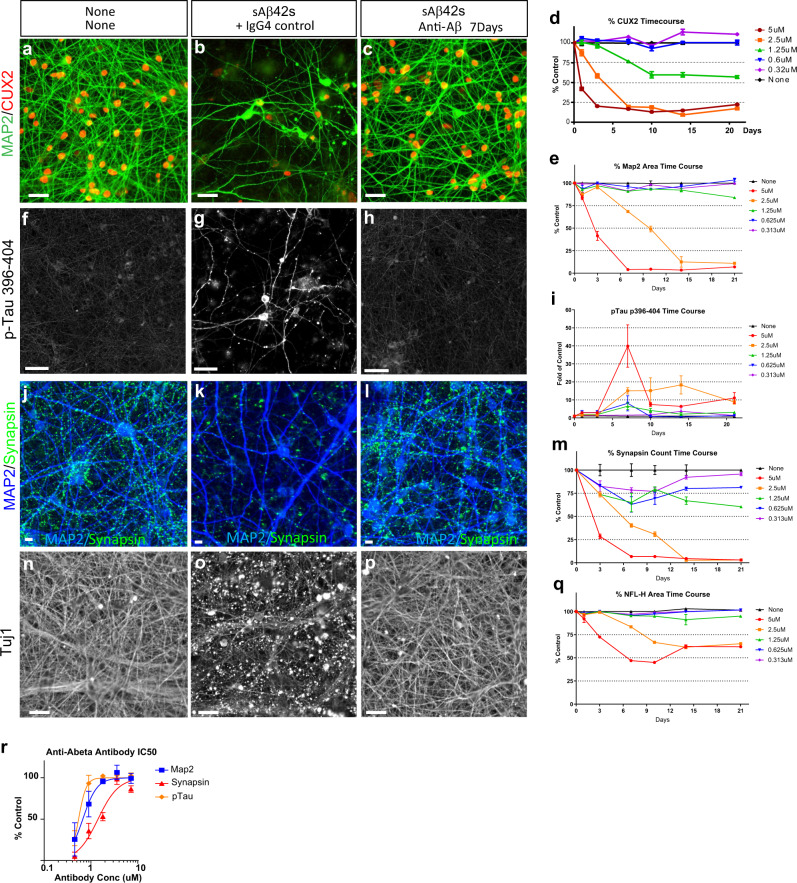
Human iPSC-derived neuronal model of Alzheimer’s disease recapitulates key hallmark AD pathologies. **a**, **b** Differentiated NAG neurons (12 weeks+) show loss of dendrites (MAP2, green) and cell bodies (CUX2, red) when treated with soluble Aβ species for 7 days (**b**) in comparison to no treatment condition (**a**). **c** Anti-Aβ antibody co-treatment with soluble Aβ species blocks Aβ-induced cell death. Scale bar 50 μm. **d** Dose-dependent, progressive cell death as quantified by the percentage of cell body (CUX2) numbers in Aβ-treated normalized to untreated control. **e** Dose-dependent, progressive dendritic (MAP2) loss as quantified by the percentage of MAP2 area in Aβ-treated normalized to untreated control. **f**, **g** Aβ42 treatment induces phosphorylation of Tau (p-Tau 396–404, white) and mislocalization to the cell body. **h** Anti-Aβ antibody co-treatment with sAβ42s blocks Aβ-induced Tau hyperphosphorylation. Scale bar 50 μm. **i** Dose-dependent and time course of phosphorylation of Tau at S396/404. Phospho-Tau induced increase at 5 µM Aβ treatment before decrease associated with cell death occurred as quantified by fold p-Tau 396/404 staining in Aβ-treated normalized to untreated control. **j**, **k** Aβ42 treatment causes synapse loss in neurons (synapsin, green). **l** Anti-Aβ antibody co-treatment with sAβ42s blocks synapse loss phenotype. Scale bar = 5 μm. **m** Dose–response and time course of synapse (synapsin 1/2) loss in Aβ-treated culture normalized to untreated control. **n**, **o** sAβ42s treatment induces axon fragmentation (beta-3 tubulin Tuj1, white). **p** Anti-Aβ antibody co-treatment blocks axon fragmentation. Scale bar = 50 μm. **q** Dose–response and time course of axon fragmentation as quantified by percentage of the axon (NFL-H) area in Aβ-treated normalized to untreated control. **r** Anti-Aβ antibody rescues all three markers in a dose-dependent manner and IC50 curves can be drawn and calculated (IC50 curve fitted by Prism software). Data are presented as mean values +/− SEM and *n* = 4 wells. All scale bars = 50 μm.

Neurons incubated with 5 μM sAβ42s show marked loss of synapses, dendrite reduction, axon fragmentation, induction of Tau hyperphosphorylation (S396/404), and dramatic cell death at 7 days (Fig. 2a–q). Several additional Tau phosphorylation sites observed in AD, S396/404, S217, S235, S400/T403/S404, and T181, were hyperphosphorylated when treated with Aβ42 oligomers (Supplementary Fig. 2V–Z). We further found that through repeated treatment of 300 nM sAβ42s soluble species for 3 weeks, we observe increased total tau (HT7) in the Sarkosyl insoluble fraction that are 3R-repeat positive (Supplementary Fig. 2Z). At this age, the iPSC-derived neurons are negative for 4R-repeat tau. Interestingly, we observed both tau fragmentation as previously reported in human AD^33^, and faint higher molecular tau bands in the Sarkosyl insoluble fraction suggesting the formation of detergent-insoluble higher molecular weight tau aggregates. Neurotoxicity was blocked by co-treatment with anti-Aβ antibody in a dose-dependent manner, indicating that pathological hallmarks of AD observed in our in vitro human neuron model are Aβ-specific (Fig. 2c, h, l, p, r). Using this quantitative platform, we generated the half-maximal inhibitory concentration (IC50) for anti-Aβ antibody rescue of MAP2, synapsin, and pTau induction (Fig. 2r). We found that synapse rescue is linear, whereas the MAP2 and p-Tau induction rescues are more indicative of a thresholded response with sharp transitions. Furthermore, the IC50 of synapse rescue is ~1.4 μM at 5 μM soluble Aβ42 species suggesting a stoichiometric relationship between anti-Aβ antibodies and sAβ42s resulting in a required 1:2 molar ratio is necessary for complete blocking.

**Fig. 3:**
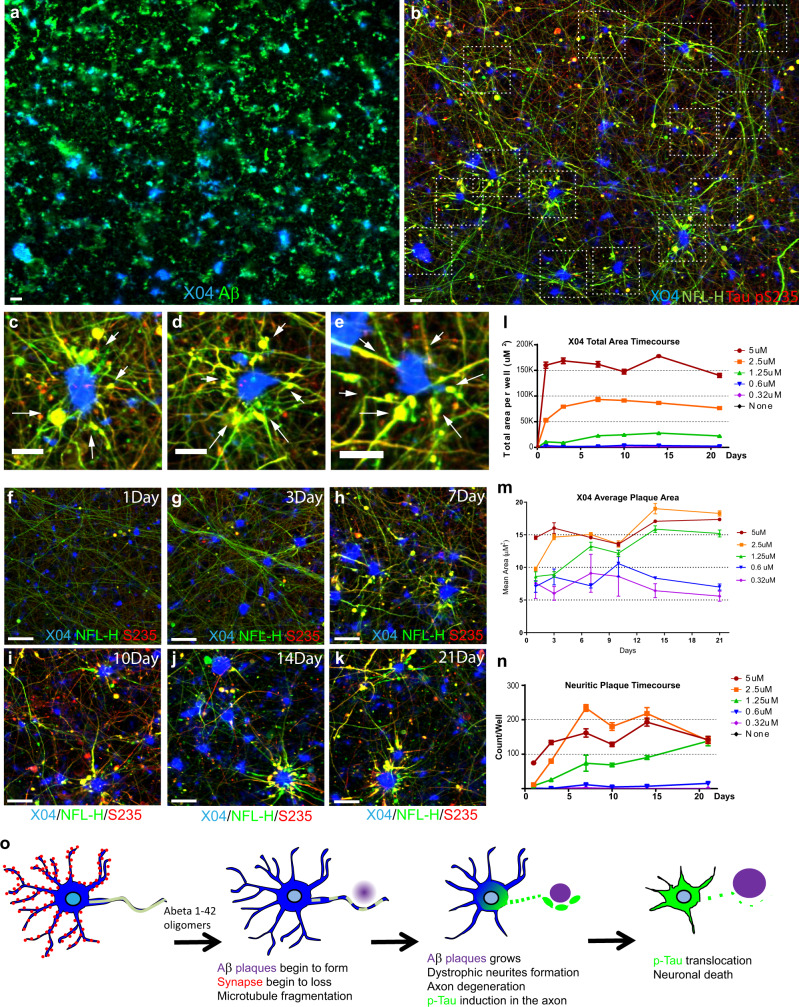
Human iPSC neurons form Aβ plaques that are surrounded by dystrophic neurites. **a**, **b** iPSC-derived neurons and primary astrocytes were treated with 2.5 μM sAβ42s for 7 days and stained for Aβ-plaque structures (**a**) (Methoxy-X04; blue), 6E10 (Aβ; green) (**b**) axons (NFL-H; green), and p-Tau (S235; red). **b** Neuritic plaques are indicated by dotted white boxes. **c**–**e** Zoomed-in images of B showing axonal swelling (NFL-H; green) and p-Tau induction (S235; red) in axons around Aβ-plaque structures (Methoxy-X04; blue). **f**–**k** Neurons were treated with 2.5 μM sAβ42s and analyzed over a 21-day time course for axonal fragmentation (NFL-H; green), p-Tau induction (S235; red), and plaque formation (Methoxy-X04; blue). Dystrophic neurites composed of NFL-H and p-Tau swellings surrounding X04-positive Aβ-plaques were observed. **l**, **m** sAβ42s were added to neuronal cultures at concentrations of 5 μM (red), 2.5 μM (orange), 1.25 μM (green), 0.6 μM (blue), and 0.32 μM (purple) on day 0, and neurons were subsequently fixed at day 1, 3, 7, 10, 14, and 21 and stained for various markers. Plaque formation (methoxy-X04-dye-positive regions) begins early after Aβ oligomer treatment and total plaque area (**l**) increases with high Aβ oligomer concentrations over time and reached maximum area at 7 days. **m** Average plaque area follows a similar trend, where average plaque size increased to around 15–20 μm and stabilized. **n** Neurons exhibit dystrophic neurite formation (measured by S235 p-Tau and NFL-H positive axon area) and these neuritic plaques increases in number with high Aβ oligomer concentrations and over time. **o** Schematic showing a summary of the observed sequential events of neurodegeneration, plaque, and dystrophic neurite formation. Data are presented as mean values +/− SEM and *n* = 4 wells. All scale bars = 50 μm.

To characterize the kinetics and the effects of soluble Aβ42 species on neurotoxicity, we performed a 21-day time course experiment with single doses of increasing sAβ42s concentrations. We observed that sAβ42s-neurotoxicity phenotypes were dose-dependent and progressive; higher doses resulted in faster pathology development and neuronal loss (Fig. 2d, e, i, m, q). The most sensitive and earliest phenotype to appear is synapse loss; a 25% reduction in synapses at 0.3 μM sAβ42s, while other neurodegenerative markers were unaffected (Fig. 2d, e, i, q). With minimal synapse damage, we observed that neurons could recover after 21 days. Interestingly, the neurotoxic response of dendrite and axon reduction to the sAβ42s has a threshold effect, whereby at 1.25 μM there is no effect on dendrite or axon reduction even though there is a sustained loss of synapses and CUX2 nuclear expression (Fig. 2d, e, m, q green line). We also observed that pTau induction seemed to be more proximal to neuronal death, as we were unable to capture the initial induction of pTau when neurons died rapidly at high sAβ42s concentrations. These findings were also recapitulated in a second iPSC-derived neuron lines (Supplementary Fig. 3), indicating the robustness of these phenotypes. These data show that our model not only exhibits hallmarks of human AD pathologies in response to soluble Aβ42 species, but also revealed a sequence of degeneration events, beginning with synapse loss, axon fragmentation, and dendritic atrophy, followed by p-Tau induction resulting in a severe neuronal loss (Fig. 3o).

In response to CNS damage and neurodegeneration, astrogliosis can often be observed and is commonly characterized by pronounced structural changes in astroglia that result in upregulation of glial fibrillary acidic protein (GFAP) and have been shown as a potential serum biomarker for AD^34,35^. To determine whether we observe this pathology, we began by demonstrating that our human astrocyte cultures express multiple astrocyte markers such as GFAP, vimentin, ALDH1L1, and EEAT1 in the characteristic astrocyte morphology (Supplementary Fig. 4A–C). After extended culturing with human iPSC neurons, we observe increasingly elaborated astrocyte processes (Supplementary Fig. 4D). We found that in response to sAβ42s, human astrocytes show a 2–3-fold elevation in GFAP expression both in monoculture and in coculture with neurons (Supplementary Fig. 4E–J). We also observed increased GFAP fragmentation (Supplementary Fig. 4G & J), which we hypothesize might represent injured or dying astrocytes, as GFAP has been shown to be cleaved by caspases during CNS injury^34^.

### iPSC-derived neurons, astrocytes, and microglia recapitulate Aβ plaque formation

**Fig. 4:**
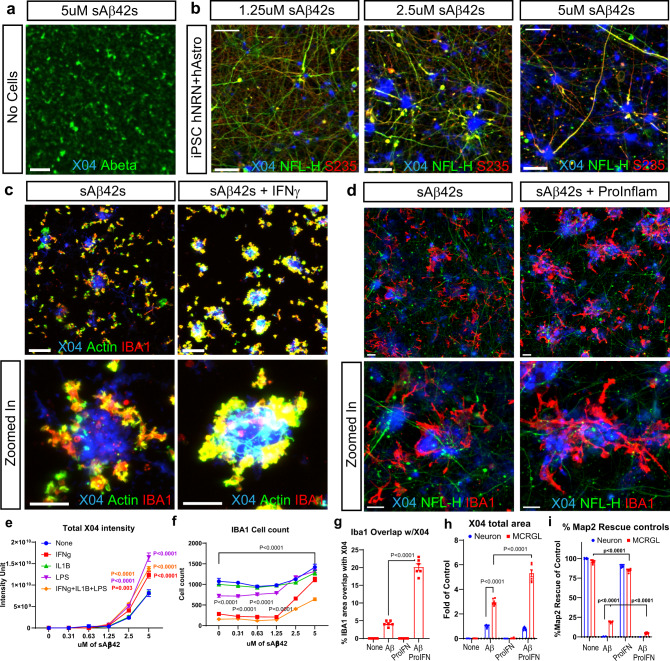
Microglia activation increases Aβ plaque formation but reduces neuroprotection. **a** sAβ42s were added to empty wells (scale bar = 20 μm) or **b** 12-week-old iPSC neurons at the indicated concentrations. All wells were stained with X04 (blue), Aβ (green), NFL-H (green), and p-Tau S235 (red). Empty wells have Aβ precipitates but no XO4 positive structure. In iPSC neuron wells, there is a dose-dependent increase of X04 staining. A Subset of XO4 is also surrounded by dystrophic neurites (NFL-H and S235 positive axonal swellings). Scale bar = 50 μm. **c** Microglia treated with sAβ42s ranging from 0 to 5 μM and also treated in combination with IFNγ. The panel below shows a zoomed-in section. Aβ plaques are stained with X04 (blue), microglia are labeled with Actin (green), and IBA1 (red). Scale bar = 50 μm. IFNγ increases plaque formation and plaque interaction. **e** Quantification of X04 intensity**. f** Quantification of IBA1 number. **d** Neuron and astrocytes coculture and triculture of neurons, astrocytes, and microglia were treated with sAβ42s with or without pro-inflammatory cytokine combination (IFNy + IL1b + LPS). Representative images from indicated conditions are shown. The lower panel of zoomed images is shown. Aβ plaque was stained with X04 (blue), dystrophic neurites swellings were stained with NFL-H (green), and microglia were labeled with IBA1 (red). In triple culture, sAβ42s addition led to Aβ plaque formations surrounded by dystrophic neurites and encircled by microglia similar to plaque presentation in vivo. Scale bar = 20 μm. **g** The area of IBA1 overlap with X04 is quantified. Pro-inflammatory cytokines increased microglia association with plaque. **h** Microglia increased the X04 plaque area, as measured by the total area of X04 staining. Pro-inflammatory cytokine addition increased the plaque area furthermore. MAP2 areas were also quantified in (**i**). sAβ42s addition caused a severe reduction to neuron culture. Microglia culture provided 25% MAP2 protection from sAβ42s. This protection is lost when pro-inflammatory cytokine is added. Data are presented as mean values +/− SEM and *n* = 4 wells. Two-way ANOVA with (**e**, **f**, **h**, **i**) Tukey’s or (**g**) Dunnett’s multiple comparisons test.

After observing hallmark AD pathologies in the human neuron model upon treatment with Aβ oligomers, we also assessed whether our model could recapitulate Aβ plaque formation. In the presence of iPSC-derived neurons and primary astrocytes, we found Aβ-aggregated structures positive for Methoxy-X04, a commonly used Aβ plaque-binding small-molecule dye (Fig. 3a). To see the plaque-like structures were made by cells, we also stained the empty culture wells treated with Aβ oligomers. We observed smaller, morphologically distinct aggregates of Aβ that were X04-dye negative (Fig. 4a). These distinct aggregates are likely the result of Aβ oligomers continuing to oligomerize and fall out of solution onto the culture plates. In contrast, incubation with Hela cells with sAβ42s does not form the same Aβ-aggregated structures positive for Methoxy-X04 as we observed in human iPSC neurons (Supplementary Fig. 6).

Further characterization showed that a subset of X04-positive Aβ plaque-like structures was surrounded by dystrophic neurites marked by neurofilament heavy chain (NFL-H) axonal swelling and phosphorylated Tau (S235) positive blebbing (Fig. 3b, c, d, e). These structures are very similar to Aβ plaques with neuritic dystrophy observed in human AD post-mortem brain sections. Importantly, neuritic plaque-like structures were also observed in the second iPSC-NSC line-derived neurons (Supplementary Fig. 3), indicating the robustness of these phenotypes. Neuritic plaques are also positive for ApoE localized in the amyloid plaques and APP in the membranes of dystrophic neurites^36,37^. We also found these two markers in vitro AD neuritic plaque-like structures (Supplementary Fig. 3C, D). To further characterize this finding, we performed a time-course experiment with increasing concentrations of sAβ42s. Time-course analysis showed that individual Aβ plaques grew in size and then peaked over 7 days (Fig. 3f–l). The growth of Aβ plaques was accompanied by the emergence of dystrophic neurite marker morphology 3 days after plaque formation which worsened over time, indicating that neurons might form dystrophic neurites as a reaction to direct Aβ plaque exposure (Fig. 3f–n). Interestingly astrocyte monocultures are also reactive to soluble Aβ species, and formed large X04-positive Aβ structures. They are large and fibrous (Supplementary Fig. 4E) and not of the characteristic compacted neuritic plaque morphology. Taken together, the data suggest that Aβ42 soluble species, in the presence of neurons and astrocytes, lead to X04-positive neuritic plaque formation resulting in neuritic dystrophy.

### Human iPSC-derived microglia lose neuroprotection in a neuroinflammatory context

Another important component of AD pathology is understanding microglial behavior in the AD disease state. Since Aβ plaques observed in human AD are often surrounded by microglial cells in a neuroinflammatory environment, we sought to address whether iPSC-derived human microglia alone could generate and surround Aβ plaques and whether neuroinflammatory cytokines modulated microglial behavior. iPSC-derived microglia used in this study expressed known markers and exhibited typical ramified morphology (Supplementary Fig. 5). They were also capable of generating and surrounding X04-positive Aβ plaques in vitro in a dose-dependent manner (Fig. 4c, e). iPSC-derived microglia stimulated with pro-inflammatory cytokines interferon-gamma (IFNγ; 50 ng/ml), interleukin 1β (IL-1β; 100 ng/ml), and lipopolysaccharide (LPS; 50 ng/ml) demonstrated increased plaque formation as measured by total X04-positive area and intensity, and surrounded Aβ plaques more closely (Fig. 4c, e). Furthermore, we observed an increase in microglial cell number, as measured via ionized calcium-binding adapter molecule 1 (IBA1)-positive cell count, suggesting a microgliosis response (Fig. 4f).

After confirming that iPSC microglia demonstrated behavior similar to in vivo observations, we proceeded to coculture microglia with our neuron–astrocyte AD model conditions and added inflammatory cytokines to understand cell–cell dynamics in an inflammatory, Aβ-neurotoxic environment. In triculture, we observed neuritic plaque formation with surrounding microglia similar to that observed in human AD post-mortem brain sections (Fig. 4d). The addition of microglia to the coculture system conferred a ~25% basal protection to neuronal health and formed threefold more Aβ plaques, suggesting that Aβ plaque formation and compaction may be neuroprotective (Fig. 4h, i). When pro-inflammatory cytokines and Aβ42 oligomers were added to the triculture system, we observed an increase in microglial-plaque association and sixfold more plaque formation, but a loss of neuroprotection (Fig. 4d, g–i). This suggests that microglial activation in response to Aβ may be beneficial in plaque compaction and neural protection, but over-activation could counteract these benefits through toxic microglial activities such as cytokine secretion.

These findings show that iPSC-derived neurons and microglia are capable of successfully modeling neuritic Aβ plaque formation surrounded by pTau positive dystrophic neurites, and encircled by microglia in close contact with the plaque; a key hallmark of AD pathology. These effects become exacerbated in a neuroinflammatory state, similar to that observed in late-stage human AD pathology. Next, we explored three potential utilities of this improved humanized AD model. We first used this system to conduct proof-of-concept focused small-molecule drug screens to further validate the model and demonstrate the screen ability of the platform. Next, we further studied the cellular process of microglial-plaque formation. And finally, we investigated the mechanism of action of a large molecule therapeutic, anti-Aβ antibody and further optimized the model to use eightfold-less sAβ42s to simulate AD progression and evaluate anti-Aβ antibody intervention.

### Focused small-molecule screen identifies DLK-JNK-cJun pathway inhibition protects human neurons from Aβ oligomer toxicity

**Fig. 5:**
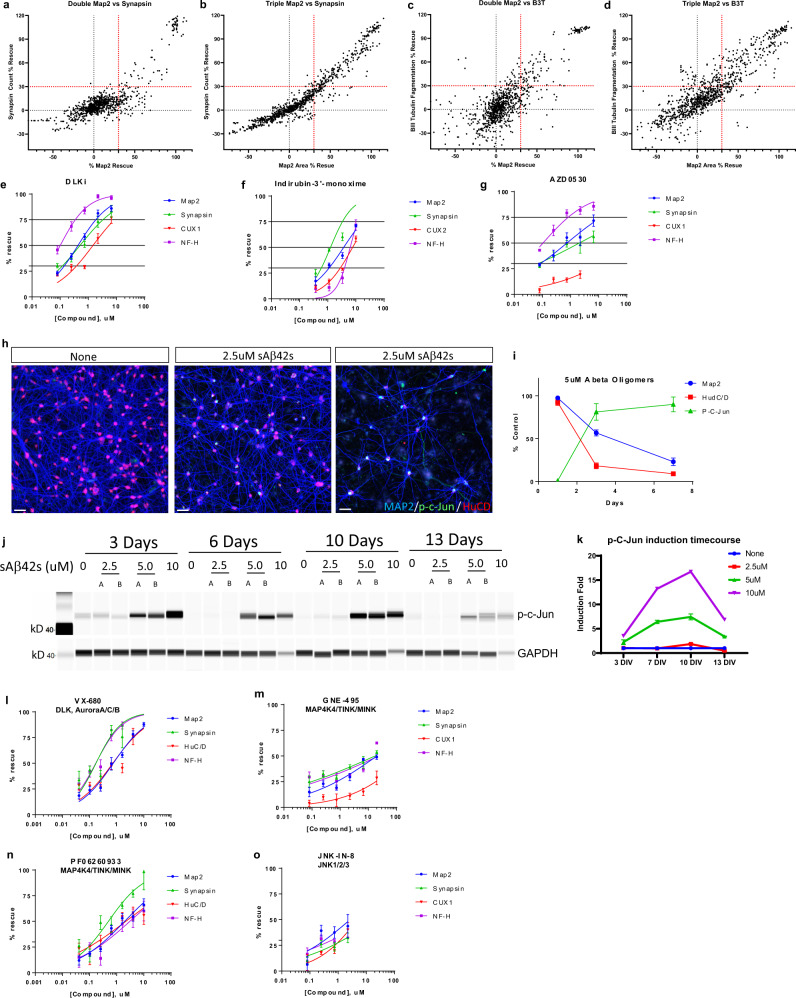
Focused small-molecule screen identifies DLK-JNK-cJun pathway inhibition protects human neurons from Aβ oligomer toxicity. **a**–**d** Neurons and astrocytes (**a**, **c**) or neurons, astrocytes, and microglia (**b**, **d**) were treated with 5 μM sAβ42s and small molecules from a focused screen of known neuroprotective agents at multiple concentrations (50, 25, 12.5, and 6.25 μM (double culture), 50, 12.5, 3.1, and 0.78 μM (triple culture). Results were graphed as Synapse % rescue versus MAP2 % rescue (**a**, **b**) and Beta III tubulin % rescue versus MAP2 % rescue (**c**, **d**). Small molecules that prevented toxicity in dendrites (MAP2), synapses (Synapsin 1/2), cell count (CUX2), or axons (NFL-H) at or above 30% were considered hits (red dotted line); anti-Aβ antibody used as a positive control that prevented all types of toxicity. **e**–**g** Further validation of top hits DLKi (**e**), Indirubin-3′-monoxime (**f**), and AZD0530 (**g**) from the focused screen by IC50 curves against MAP2 (blue), Synapsin 1/2 (green), CUX2 (red), and NFL-H (purple). **h** sAβ42s treatment induced expression of p-cJun (green) in the nucleus (HuCD, red). **i** Quantification of MAP2 (blue), HuC/D (red), p-cJun (green) staining indicated an increase in cJun phosphorylation with prolonged sAβ42s treatment. **j** 22-week-old iPSC neuron culture treated with sAβ42s showed dose-dependent, sustained phosphorylation of cJun as shown by western blot. GAPDH served as the loading control. **k** Western blot quantification of p-cJun induction normalized to GAPDH. **l**–**o** Inhibition of known components of DLK-JNK-cJun pathway using small molecules VX-680 (**l**), GNE-495 (**m**), PF06260933 (**n**), JNK-IN-8 (**o**) prevents sAβ42s-induced neural toxicity in all measured markers in a dose-dependent manner. Data are presented as mean values +/− SEM and *n* = 4 wells (**e**–**g**, **I**, **k**–**o**). IC50 curves fitted by Prism software (**e**–**g**, **m**–**o**).

To demonstrate a proof of concept of screen ability and investigate whether observed AD pathology preserved molecular signaling events previously demonstrated in AD, we conducted a focused screen of 70 small molecules that have previously been shown to confer neuroprotection in various neurotoxic contexts in addition to AD (Supplementary Table 1). In double and triple cultures, we tested these small molecules in our AD model paradigm at four concentrations as different molecules could have different effective concentrations. We considered hits as molecules that characterized ≥30% rescue in any of the four measurements of dendrites area (MAP2), synapses count (Synapsin 1/2), or cell count (CUX2), or axon area (beta III tubulin) (Fig. 5a–d, Supplementary Table 2, and Supplementary Table 3). We see overlapping hits from double and triple cultures which are the most promising top hits. We confirmed nine hits from both screens with IC50 curves in double culture, including inhibitors of well-known active kinases in AD such as DLKi^27,38^, Indirubin-3′-monoxime (GSK3β and CDK5 inhibitor^26^), and AZD0530 (Fyn inhibitor^25^). Importantly, GSK3, CDK5, and Fyn are known Tau-acting kinases^25^. We also followed up on two natural products, luteolin, and curcumin, which have shown to provide a protective effect in AD^39^. Curcumin and its derivative, J147, were validated with IC50 curves^40^ (Supplementary Fig. 7). Curcumin and J147 are currently undergoing investigation in Phase I and Phase II clinical trials, respectively^41,42^. In addition, we identified multiple calpain inhibitors from the primary screens and validated Demeclocycline HCl with an IC50 curve (Fig. 5e–g, Supplementary Fig. 7, Supplementary Table 2, Supplementary Table 3). Calpains were previously identified to be active in AD^43^, and have been implicated in CDK5 signaling and Tau cleavage.

Since DLK inhibition was the most protective compound, and we also see JNK inhibition (AS601245) was also protective to a less extend in our focused screens in both double and triple cultures, we sought to validate this pathway and investigate whether the DLK-JNK-cJun signaling pathway is activated in our AD model. DLK is uniquely expressed in neurons^44^, we only focused on neuron–astrocyte co-cultures. Phospho-cJun has been previously observed in human AD brain tissue and mouse models as a readout of DLK pathway activation^38^. We observed induction of the phosphorylation of cJun when human neurons were treated with Aβ42 oligomer (Fig. 5h, i). This effect was persistent (up to 13 days) and increased in a dose-dependent manner with soluble Aβ42 species concentration (Fig. 5h–k).

To further validate this pathway, we tested several known inhibitors of kinases in the DLK signaling pathway to determine whether they could also be neuroprotective in our AD model. We observed that inhibition with VX-680 (a different DLK inhibitor), GNE-495 (MAP4K4 inhibitor upstream of DLK^45^, PF06260933 (a different MAP4K4 inhibitor), and JNK-IN-8 (JNK1/2/3 inhibitor) conferred neuronal protection against Aβ in a dose-dependent manner (Fig. 5l–o).

Our focused screen resulted in the identification and validation of several compounds targeting proteins in several known mechanisms in human AD such as DLK, GSK3, CDK5, and Fyn kinase, all of which are current pathways of interest in drug development. This shows that our in vitro human neuron-based AD model, not only demonstrates phenotypes of AD not previously seen in vitro, but also recapitulates important pathological signaling events that contribute to these observed phenotypes. In sum, the validation of known molecular signaling pathways previously shown to be important drug targets suggests that our in vitro human neuron AD model is a translationally relevant molecular neurobiology and high-throughput screening tool that can facilitate target discovery and characterization and larger drug development efforts.

### Cellular mechanism of microglia amyloid plaque formation

**Fig. 6:**
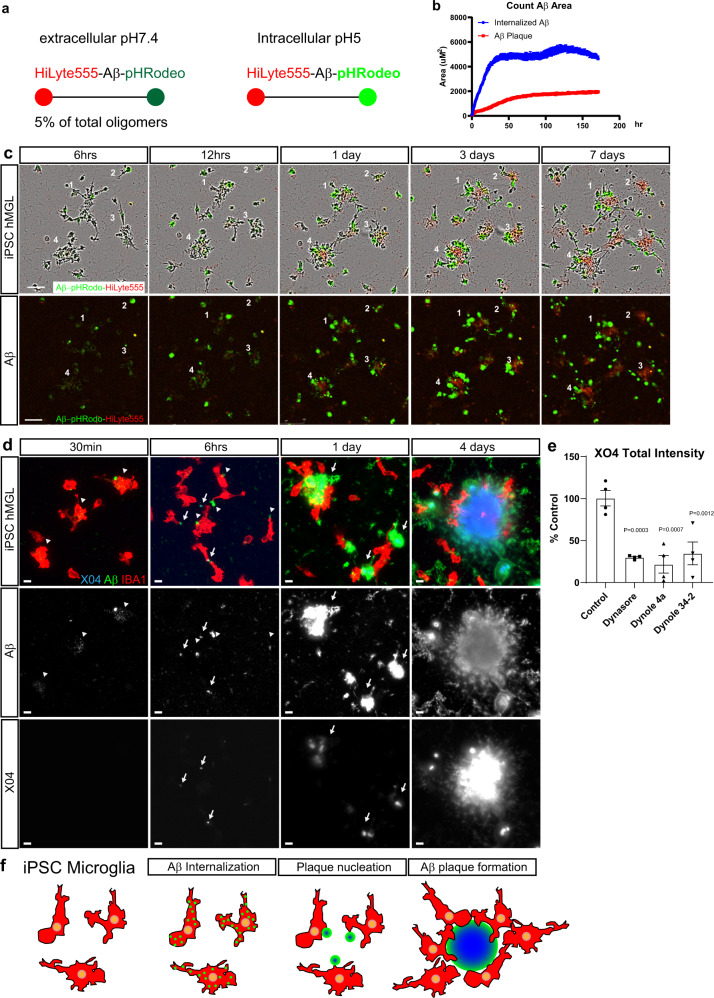
Human iPSC microglia internalizes soluble Aβ species to form extracellular plaques. **a** Schematic showing sAβ42s labeled by HiLyte-555 and pHrodo Green continuously fluoresce red, but only fluoresce green under intracellular pH 5 conditions. **b** Quantitative analysis of red Aβ plaque area and green internalized Aβ. Internalized green Aβ outpace the red extracellular Aβ plaque formation, indicating active Aβuptake throughout the 7 days and proceed before the appearance of red Aβ plaques. **c** Example images of the plaque formation time-lapse movie. Four different plaque formations are retrospectively labeled. sAβ42s are first internalized by microglia (green) before plaque formation (red) in the center of the cultured microglia. Scale bar = 100 μm. **d** iPSC-derived microglia were treated with 5 μM sAβ42s, then fixed and stained after 30 min, 6 h, 1 day, and 4 days. Microglia (IBA1, red) internalize small Aβ puncta (green; white—the second row) indicated by white arrowheads (green) after 30 min, then externalize these puncta as large aggregates that are faintly X04 positive (blue; white—bottom row) indicated by white arrows, then form large, extracellular X04-positive plaque structures surrounded by microglia from 1 to 6 days. **e** Human iPSC-derived microglia were treated with 5 μM soluble Aβ species and various dynamin inhibitors (Dynasore, Dynole 4a, Dynole 34-2) at 0.6 μM for 24 h and plaque-like structures (Methoxy-X04-positive) were quantified as a percentage of control. Treating with dynamin inhibitors decreased plaque formation approximately fourfold in all conditions. **f** A summary of steps of microglia plaque formation observed. Data are presented as mean values +/− SEM and *n* = 4 wells. One-way ANOVA with Dunnett’s multiple comparisons test.

Since our AD model system recapitulated amyloid plaque formation robustly, we thought to understand the cellular process of plaque formation. To observe plaque formation by microglia, we conducted time-lapse studies at 30 min intervals for 7 days with microglia and compared to a similar cell type: human CD14-derived macrophages (Supplementary Movies 1, 2). HiLyte-555-labeled Aβ42 monomers were used to generate red soluble Aβ42 species (Supplementary Fig. 8A). Microglia were uniquely highly motile during and after plaque formation compared with the macrophages, extending and retracting their processes and moving dynamically in and out of plaques (Supplementary Movie 1 and Supplementary Fig. 4C). Aβ plaque formation appeared to form extracellularly within clusters of microglial cells and grew larger (Supplementary Movie 1 and Supplementary Fig. 8B). In contrast, human macrophages were relatively stationary and continuously internalized red sAβ42s (Supplementary Movie 2). We then incorporated pHrodo^®^ green dye into HiLyte-555-labeled oligomers to allow for a concurrent observation of Aβ42 internalization (green) and plaque formation (red) (Fig. 6a, Supplementary Movie 3). We found that microglia first internalized sAβ42s preceding plaque formation (Fig. 6b, c, Supplementary Movie 3). These results taken together, suggest that microglia may internalize soluble Aβ42 species first, and then exocytose and package them as plaque structures (Supplementary Fig. 6E).

To further confirm the time-lapsed result, we performed an immunostaining time-course study. We observed microglia took up sAβ42s within 30 min (Fig. 6d). After 6 h, small internalized puncta disappeared and a larger, faintly X04 positive, Aβ42 aggregate appeared at the edge near each cell (Fig. 6d). After 1 day, larger Aβ42 aggregates with higher X04 staining intensity were seen next to microglia, and additional microglia began to surround these aggregates. At 4 days X04-dye-positive plaque structures were present with surrounding microglia. This behavior appeared unique to microglial cells, as human macrophages appear to continuously internalize sAβ42s, and then appear to die (Supplementary Fig. 9). Finally, to test if endocytosis is involved, microglia were treated with dynamin inhibitors which reduce endocytosis. We found that there is a 75% reduction of plaque formation indicating that microglia internalization of Aβ42 is critical for amyloid plaque formation.

### Modeling AD progression and anti-Aβ antibody intervention

**Fig. 7:**
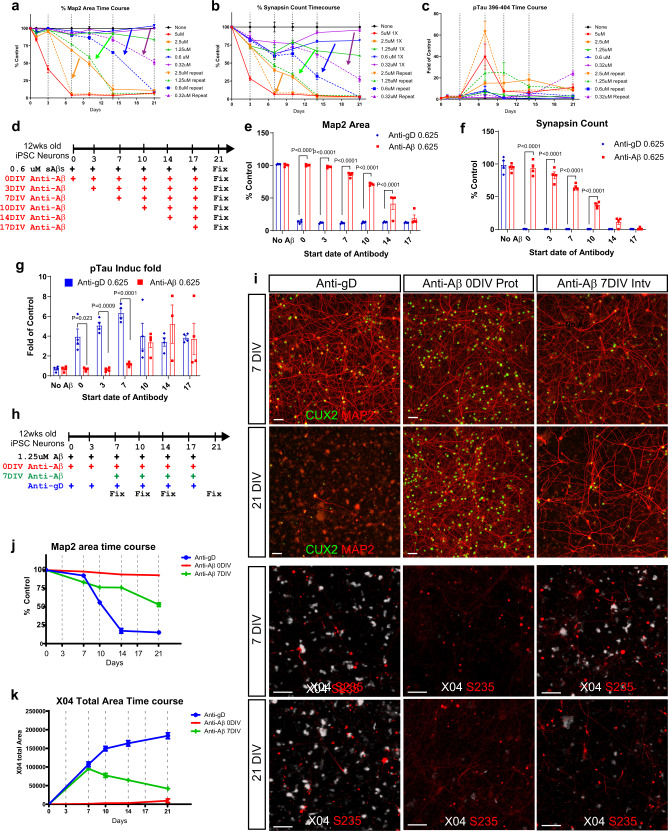
An early window of efficacy for anti-Aβ antibody revealed by disease progression modeling. **a**–**c** Time-course comparison of 12-week-old iPSC neurons treated with a single dose of sAβ42s (solid lines) versus repeated dose of sAβ42s at the same concentration (dotted lines) at the indicated concentration. MAP2 area (**a**), Synapse count (**b**), and p-Tau 396–404 induction fold (**c**) were quantified. **d** Repeated dosing schedule of 12-week-old neuron with 0.6 μM of Aβ. Anti-Aβ antibodies dosing regimen was started at indicated time point. All cells were treated in the same plate and fixed at 21 days post first dose. MAP2 area (**e**), synapsin count (**f**), and p-Tau induction fold (**g**) were quantified. Anti-gD antibodies were dosed similarly as control (blue bars) along with anti-Aβ antibody (red bar). **h** Time-course study design of Anti-Aβ antibodies repeated dosing. sAβ42s are added at every indicated timepoints. Anti-Aβ antibodies were added at day 0 (red) as a protection model or at day 7 (green) as an intervention model. Anti-gD antibodies were used as control (blue). **i** Representative images from the indicated experimental treatments. Neurons were stained for dendrite marker MAP2 (red) and nuclear marker CUX2 (green) at 7DIV and 21DIV. The lower panel shows Aβ plaque staining (X04, white) and p-Tau S235 (red) staining. Quantification of MAP2 area over time (**j**) and plaque area (**k**) illustrated that the anti-Aβ intervention model is capable of slowing down neuron degeneration and plaque formation. Scale bars = 50 μm. Data are presented as mean values +/− SEM and *n* = 4 wells. Two-way ANOVA with Tukey’s multiple comparisons test.

A high single dose of sAβ42s (5 μM) while convenient to show sAβ42S phenotypes within 7 days, might not be as physiological, where it is likely there is a lower elevation of Aβ42 oligomers over an extended time. To model the progression of AD and continuous Aβ exposure, we systematically added repeated doses of Aβ oligomers to neuron/astrocyte cultures twice a week after media changes at several concentrations (0.3–5 μM) throughout a 21-day time-course study. Repeated low dosing of Aβ42 oligomer led to prolonged, increased neuronal toxicity compared with a single exposure (Fig. 7a–c, solid vs dotted line). We chose repeated doses of 0.625 μM to model AD progression, which took 21 days to cause cell death.

As observed earlier, anti-Aβ antibodies were protective at high concentrations when added at the onset of Aβ exposure (prophylactically). However, in clinical settings, some degree of neuronal damage would have already occurred by the time of therapeutic intervention. We tested whether anti-Aβ antibody treatment is effective when Aβ-induced neurotoxicity has occurred before treatment by creating an antibody intervention model where anti-Aβ treatment began after various lengths of sAβ42s exposure (Fig. 7d). We found that there is a window, around two-thirds of the way through the disease progression course, where anti-Aβ antibody treatment could provide neural protection in dendrites, synapses, and pTau induction (Fig. 7e–g, Supplementary Fig. 10). Interestingly, we saw that the window of protection against pTau induction is shorter than that of synapsin (7 versus 14 days, respectively), suggesting that anti-Aβ antibodies may be most effective before pTau induction. Furthermore, the intervention window is shortened when neurodegeneration is sped up by using an increasing amount of sAβ42s per bi-weekly dose (Supplementary Fig. 10).

Next, we conducted a time-course analysis of the MAP2 area as a measure of neuronal health, Methoxy-X04 staining for plaque formation, and pTau (S235) as a measure of pTau induction and dystrophic neurites (Fig. 7h–k). We found that anti-Aβ antibodies reduced the progression of neurodegeneration and plaque formation compared with the anti-gD control antibody (Fig. 7i–k). These data demonstrated that our AD model can be modulated to generate a progressive AD disease model with precise temporal control of neurodegeneration speed. We evaluated the neuroprotective capabilities of anti-Aβ antibodies and demonstrated that earlier intervention confers greater protection, in line with the current consensus in the field.

### Anti-Aβ antibodies protect neurons by keeping Aβ oligomers in soluble supernatant

**Fig. 8:**
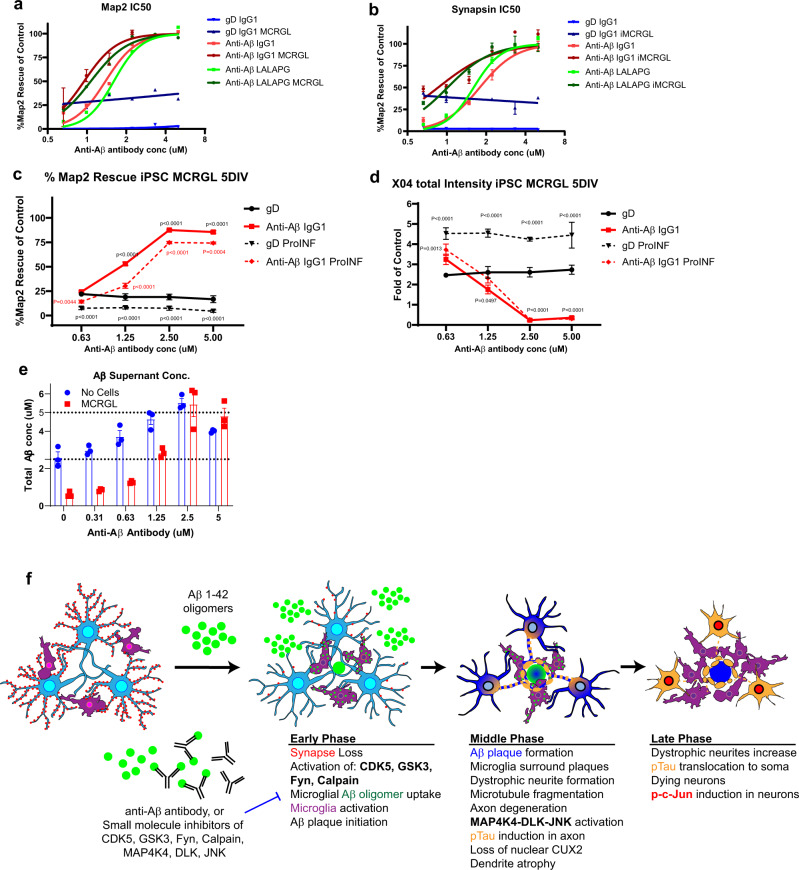
Anti-Aβ protects neurons by keeping Aβ soluble in supernatant and antibody effector function is not required. **a**, **b** iPSC neurons and astrocytes treated with 5 μM sAβ42s followed by serial dilutions of anti-gD and anti-Aβ antibodies with IgG1 and LALAPG backbones with and without iPSC microglia. Results for dendrite protection (MAP2 area) (**a**) and synapse protection (synapsin count) (**b**) were analyzed via IC50 curve fitting using Prism software. Microglia provide baseline protection as shown by an upward shift in the anti-gD graph when microglia are added (gD IgG1 alone, blue; gD IgG1 + microglia, dark blue). Anti-Aβ antibody backbones protect dendrites and synapses similarly without microglia (Anti-Aβ IgG1, red; Anti-Aβ LALAPG, green) and with microglia (Anti-Aβ IgG1, dark red; Anti-Aβ LALAPG, dark green). **c**, **d** Neuron, astrocyte, microglia triculture treated with 5 μM sAβ42s (solid lines) and pro-inflammatory cytokines (dashed lines), then serial dilutions of gD antibody (black lines) and anti-Aβ antibody (red lines) were added. **c** Basal dendrite protection (MAP2 area) is lost in a neuroinflammatory environment, and anti-Aβ shows dose-dependent efficacy. **d** Plaque formation (Methoxy-X04 total intensity) increases in pro-inflammatory conditions, however, anti-Aβ shows similar plaque reduction. **e** iPSC microglia (red) were treated with 5 sAβ42s and serial dilutions of anti-Aβ antibody; no cells wells were used as control (blue). The supernatant was collected and anti-Aβ ELISA was performed to measure total Aβ concentration. Anti-Aβ antibody treatment increases soluble Aβ species present in the culture supernatant. **f** Summary of sequential events in the iPSC AD model. Data are presented as mean values +/− SEM and *n* = 4 wells. Two-way ANOVA with Tukey’s multiple comparisons test.

We sought then to address how anti-Aβ antibodies confer neuronal protection in a complete triculture system with microglia, neurons, and astrocytes. We treated our triculture model with Aβ42 oligomers and several anti-Aβ antibodies and gD controls with different effector functions: Immunoglobulin G1 (IgG1; high effector function) and effector-less (LALAPG) antibodies^46^. Antibody IC50 was calculated as a measure of neuronal protection. We examined anti-gD antibodies in the presence or absence of microglia to understand microglial baseline protection and observed ~25–40% protection of neural synapses and dendrites (Fig. 8a, b). Anti-Aβ antibodies showed increased protection as well as in the presence of microglia, suggesting that microglial neuroprotection and anti-Aβ antibody protection are additives (Fig. 8a, b). Comparisons with antibodies with an effector or effector-less function revealed no significant difference, suggesting antibody effector function may not play a role in this model.

To determine whether a neuroinflammatory environment affects antibody protection in the presence of microglia, we added pro-inflammatory cytokines IFNγ (50 ng/ml), IL-1β (100 ng/ml), and LPS (50 ng/ml) to activate microglia and measured neuronal health (MAP2) and plaque formation (Methoxy-X04). The control, anti-gD antibody, replicated our previous observations (Fig. 5g–i) that microglial neuroprotection was lost in a neuroinflammatory state (Fig. 8c). Interestingly, we found that anti-Aβ antibodies are protective in both contexts, with the IC50 curve shifting to the right presumably due to the loss of microglial protection (Fig. 8c).

Since the presumed mechanism of action of anti-Aβ antibodies is to bind Aβ, we hypothesized that sAβ42s remain solubilized in the supernatant, bound to antibodies, and neutralized from causing toxicity to neurons in this state. We analyzed the supernatant containing 5 μM sAβ42s with increasing antibody concentrations and found that anti-Aβ antibodies increased soluble Aβ in the supernatant and decreased plate-bound Aβ (Fig. 8e). Soluble Aβ present in the supernatant was reduced in the presence of microglia, this might be due to increased plaque formation (Fig. 4c) or microglial endocytosis and clearance. However, with increasing antibody concentrations, Aβ in the supernatant increases to the original input of 5 μM. This suggests that anti-Aβ antibodies bind and solubilize Aβ, reducing contact with neurons and microglia, thereby conferring neuronal protection independent of microglia, which is consistent with our observation that anti-Aβ antibodies treatment results in decreased plaque formation (Fig. 8d, e).

## Discussion

In summary, we have generated a human iPSC AD model comprised of human neurons, astrocytes, and microglia. In this high-throughput triple culture system, the addition of soluble Aβ42 species not only recapitulated the hallmarks of AD but also developed in a sequential order of events that is similar to human AD disease progression. Our observations are consistent with previous observations and proposed disease progression mechanisms^12,13,18^. In this in vitro iPSC AD model, in the early phase, we observed that synapse loss is the most sensitive pathology to soluble Aβ42 species exposure (Fig. 8f). Small-molecule experiments also revealed that sAβ42s trigger the activation of calpain, CDK5, GSK3, and Fyn as their inhibition provides neuroprotection. At the same time, microglia internalize sAβ42s and exocytose compacted sAβ42s and initiate external plaque formation. Astrocytes also respond to s sAβ42s and we observed an increase of GFAP level, consistent with an astrogliosis response. In the middle phase, Aβ plaque formation increases, and microglia surround the plaques. Dystrophic neurites positive for phospho-Tau also start to form around the plaque. Aβ42 toxicity reaches a threshold and elicits microtubule fragmentation and axon degeneration that may trigger further MAP4K4-DLK-JNK activation. The CDK5, GSK3, and Fyn activation continues from the early phase and leads to pTau induction that begins in the axon. The loss of nuclear CUX2 and dendrite atrophy is evident at this stage. In the late phase, we observed Aβ plaques formation reaches completion, and dystrophic neurite formation increases around the plaques. In the degenerating neurons, phosopho-tau is found in the neuronal cell body and dendrites. There is also induction of nuclear phospho-cJun. Microglia could continue to be activated and produce neuroinflammatory cytokines. We also observed that anti-Aβ antibodies can protect neurons from these pathologies when used within the intervention window. In addition, small-molecule inhibitors to activated pathways could provide varying degrees of neuroprotection.

The advances in human iPSC neuronal differentiation protocols have generated excitement due to the translational potential of human disease modeling and application for target discovery and drug screening. However, human iPSC neurons are sensitive and require extended culturing time (80+ days) to develop mature neuron characteristics^1^. This is challenging to do consistently and on a large scale by manual media changes, resulting in most small-molecule and CRISPR screenings being conducted using <30-day-old neurons^2,3,47^. Many neurodegenerative diseases are adult onset, thus we reasoned that a high-throughput screening platform using older neurons could be more translational. In addition, observing hyperphosphorylation of Tau and synapse loss is only possible using older neurons, as younger neurons transiently express high levels of phosphorylated tau and have not yet developed mature synapses, underscoring the importance of long-term culturing methods for AD research. We developed a scalable, automated human iPSC neuronal culture platform operated by robotic automation to achieve consistent and long-term neuronal health in a convenient walk-away system. We then further coupled this culturing platform with an automated cell washer and high content imager to achieve systematic and high-throughput automated image analysis with unbiased quantitative read-outs on thousands of neurons for neuronal morphology-based arrayed screens.

We expect that the technology described here can be broadly applied to other neuron-relevant assays for various neurological diseases such as ALS, FTD, autism, or Parkinson’s diseases requiring mature neurons. Homogeneous motor neurons^48,49^, inhibitory neurons^47^, and dopaminergic neurons^50^ can be generated using an inducible transcription factor approach and cultured on this platform. Additional specific neuronal subtypes are being revealed with systematic screens to uncover novel transcription factor combinations^51,52^. Thus, the generation of disease models with different neuron subtypes from the same parental iPSC and conducting arrayed high-throughput screen could uncover neuronal subtype-specific gene function and facilitate fundamental understanding of how specific neuronal subtypes are selectively vulnerable in neurodegenerative diseases.

Using the automation methods described here, we demonstrate the utility of this culturing platform and generated a human cell-based AD model in vitro using iPSC-derived neurons, astrocytes, and microglia. This AD model captures critical hallmarks of human AD pathologies—such as severe synapse loss, followed by the p-Tau induction and resulting in neuronal loss—previously absent from various mouse models overexpressing mutant APP and/or PSEN. Strikingly, this human AD model recapitulates the robust Aβ plaque formation that includes surrounding pTau positive (S235 and T181) dystrophic neurites and human iPSC microglia in close contact with the plaque, all in a convenient 2D-culture formation.

This system is consistent with the amyloid hypothesis and is capable of recapitulating AD pathological hallmarks with the addition of exogenous soluble Aβ42 species. Because of this platform’s large scale and consistent nature, the pharmacology and kinetics of the sAβ42s toxicity were precisely and systematically analyzed. Synapse loss is the most sensitive and the earliest phenotype to appear and neuron recovery is possible given time. We also found that the neurotoxic response of dendrite atrophy and axon fragmentation have a thresholded effect, such that 1.25 μM sAβ42s lead to a sustained loss of synapses and CUX2 nuclear expression but no dendritic or axonal atrophy (Fig. 2d, e, m, q, green line). Whereas at 2.5 μM there is robust dendritic atrophy and axonal fragmentation (Fig. 2d, e, m, q, orange line). Interestingly, CUX2 has been shown to be required for dendrite branching, spine development, and synapse formation in layer II–III neurons of the cerebral cortex, and CUX2 KO mice have defects in working memory^53^.

Further corroborating this thresholded neurodegeneration response, we also see this in the repeated dosing paradigm. The 1.25 μM repeated dosing group have the same time course in decline as single 2.5 μM dose after 2 exposures; and 0.625 μM repeated doing group began to rapidly decline after 4 exposures, indicating that when repeated dosing Aβ42 oligomer reached the threshold of 2.5 μM total exposure the neurons began to decline (Fig. 5a, b, orange, green, and blue dotted lines). In this system, the media were refreshed 50% at each dosing so there are <2.5 μM total sAβ42s in the culture, but neurons will have been cumulatively exposed to 2.5 μM, seemingly the threshold for the decline.

The pathogenesis of sporadic AD could be the impairment of endogenous Aβ clearance that leads to excessive Aβ overall level. Furthermore, human AD is marked by the gradual and chronic accumulation of cell-derived, pathogenic Aβ species. This modeling system was based on the amyloid hypothesis and exogenous Aβ oligomers were used as the trigger, which recapitulates most of the phenotypes in familial AD indicating that synthetic sAβ42s prep can capture similar pathogenic species. The observed variability of the different Aβ42 peptide lots in the ability and the degree in triggering neuronal pathologies could suggest that the synthetic soluble Aβ42 species that are generated from each lot of the Aβ42 peptides could have different compositions of oligomer or fibril conformational species. And only a subset is disease-causing. While we carefully selected the specific lots that best mimic the pathologies, we might not fully recapitulate the same coverage(s) of Aβ42 soluble toxic species with synthetic Aβ42 peptides as that of cell-derived soluble Aβ species. Potential optimization of this modeling system could incorporate inducible over-expressions of familial mutant APP and PSEN, and/or KI mutations, KO of known genes that affect Aβ42 clearance to better mimic the human disease condition.

We also observed that pTau induction was a thresholded response and more proximal to neuronal death. In addition, the IC50 of anti-Aβ synapse rescue is linear whereas dendrite area and pTau induction rescues appear to operate based on a threshold system (Fig. 2r). In a progressive AD model, anti-Aβ antibodies can either fully rescue or not rescue the pTau phenotype at all when treated within an early intervention window (Fig. 7g, Suplemmatary Fig. 10). These data taken together illustrate a scenario where in the early phase of AD, neurons could be repaired from low levels of Aβ42 oligomer toxicity such as synapse loss. When this prolonged, constant exposure hits a threshold of severe synapse loss, dendrite and axon fragmentation then begin. At the final stages of Aβ42 toxicity, pTau induction occurs, is no longer dependent on Aβ oligomers, and is followed by severe neuronal loss.

AD-tau pathology is under intense study, and it is composed of hyperphosphorylated tau aggregates. In Alzheimer’s disease (AD) brain pathological tau is hyperphosphorylated containing 3R and 4R-repeat isoforms and is polymerized into paired helical filaments (PHF) forming neurofibrillary tangles. Some of the tau in AD brains is truncated which also promotes its self-assembly. In a previous study, Espuny-Camacho et al.^24^ implanted human iPSC neurons into AD mouse models, after 6 months this chimeric mouse model recapitulates pTau induction and human neuronal sensitivity phenotypes and thus generated early tau pathological features but no robust tau aggregates. We are able to recapitulate similar early events of the tau pathogenesis with sAβ42s induction in vitro. In this model, we observed dose-dependent hyperphosphorylation of tau at multiple sites and the formation of detergent-insoluble higher molecular weight tau aggregates in the progressive AD model. However, the neurons in our culture are at an early age which does not express 4R-repeat Tau, consistent with the findings of Espuny-Camacho et al. work in vivo. We were unable to recapitulate the AD-tau aggregates made of 3R and 4R tau at 2–3 months of neuronal culture. To further improve this system to generate the specific AD pathogenic tau species, iPSC could be engineered to express robust 4R tau early.

As a demonstration of screen ability and validation of this human iPSC AD model, we conducted a focused screen. Nine compounds were validated. Importantly, we identified and showed that inhibition of known Tau-acting kinases such as GSK3, CDK5, and Fyn conferred neural protection from Aβ toxicity^25^. This highlights the link of Aβ toxicity to Tau in this system. Of note, the DLK-JNK-cJun pathway has also been shown to be activated in vivo in human AD brains^38^. We observed similar activation of cJun proximal to neuronal loss in our in vitro system, providing further evidence of the translatability of our model. We showed that inhibition of the MAP4K4-DLK-JNK-cJun neuronal stress pathway with multiple small molecules targeting different members of the pathway also confer neuroprotection. In conclusion, we have generated a highly translatable, human AD model capable of recapitulating hallmarks of AD pathologies ready for large-scale screening.

Human iPSC-derived microglia offers a powerful tool to study disease-associated inflammatory responses. However, microglia are highly sensitive to their environment, when cultured in vitro there is a loss of some microglial gene signatures^54^. Furthermore, rodent microglia fail to fully recapitulate the human expression pattern^55–58^. In our system, we found that iPSC microglia monoculture or in triple culture, maintain the critical function of plaque formation and this response to Aβ oligomers can be further modulated by pro-inflammatory cytokines. While there could still be some gene signature deficiencies, which warrant further analysis, it seems the core microglia plaque formation gene program is intact. Thus this AD model opens new avenues for exploration, especially when coupled with CRISPR technology to generate disease-associated genetic knockout or mutant microglia.

The role of microglia in plaque formation has long been debated. Several in vivo studies using microglia ablation have been conducted—Grathwohl et al. showed that early ablation of microglia prior to plaque onset did not prevent Aβ plaque formation, while Spangenberg et al. showed contradictory results^18,59,60^. In our in vitro model, we observed that neuritic plaques still formed robustly in the absence of microglia. However, with the presence of microglia, this process is greatly accelerated and increased threefold. This suggests that human neurons, astrocytes, and microglia all play important roles in shaping the Aβ plaque formation. Using this in vitro plaque formation assay, we found evidence that microglia internalize Aβ oligomers then export them out as extracellular Aβ fibril seeds that may promote further plaque formation. This is consistent with previous observations in the AD brain sections^61–63^. Multiple mechanisms of microglia’s role in plaque formation and its effect whether protective or damaging, have been proposed^61–63^. We found that the plaque formation process by microglia is neuroprotective, although microglial activation with inflammatory cytokines such as IFNy and LPS while enhancing plaque formation, did not provide further neuroprotection. Instead, neuroprotective effects are lost, demonstrating this in vitro model captures the complex role of microglia in AD disease progression and that context is important. Future studies with this system incorporating cytokine analysis and microglia RNAseq analysis in combination with disease-associated genetic mutant microglia could enhance our understanding in this area.

The use of exogenous Aβ oligomers gives us control over the speed and severity of disease progression. This allowed us to generate a slower progressive model of AD and potentially investigate the mechanism of action of therapeutics. Recently, several anti-Aβ therapeutics’ phase 3 trials have not been successful^18^. We investigated the MoA of anti-Aβ antibodies to understand the potential criteria for success and found that a high ~1:2 molar ratio of antibody to Aβ oligomer is required to confer neural protection. Antibody-bound Aβ remains soluble in the supernatant and prevents plaque formation. In addition, we observed a temporal window where the anti-Aβ antibody was able to slow neurodegeneration after the onset of neural damage and demonstrate that early intervention provides the greatest benefit. Notably, we show that early anti-Aβ antibody intervention can prevent further Aβ oligomer-induced Tau hyperphosphorylation and plaque formation. In addition, antibody effector functions do not appear to be required in this in vitro model. Importantly anti-Aβ antibodies maintain efficacy in the neuroinflammatory environment often present in AD. Extrapolating from these findings, this would suggest that high brain exposure and earlier treatment in the disease could be both important for the success of an anti-Aβ antibody therapeutics. Perhaps the proof of concept of the anti-Aβ approach will come from the Alzheimer’s Prevention Initiative Autosomal-Dominant Alzheimer’s Disease Trial, where anti-Aβ antibody is being administered pre-symptomatically to Columbian family carrying the PSEN1 E280A autosomal-dominant mutation that causes early-onset Alzheimer’s disease^64^. We hope to provide sufficient demonstrations of the utility of this in vitro progressive AD model and will encourage studies examining other potential therapeutic and may similarly define possible intervention window.

There are several areas for further development of our platform. Neurons, astrocytes, and microglia are highly specialized and disease-relevant cell types, and thus it is crucial to study certain human gene functions in these cells. Future incorporation of CAS9 expression into the system could allow for CRISPR screening of target discovery and/or validation for translational research. Tian et al. have recently demonstrated the use of an inducible CRISPR inhibition (CRISPRi) system in iPSC neurons to inhibit gene expression in the differentiated neuron state without interference with the differentiation process^64^. Incorporation of CRISPR activation (CRISPRa) will enable gain-of-function genetic screens complementary to CRISPRi loss-of-function screens. Future drug screening, target discovery, and mechanism of action studies utilizing this high-throughput culturing platform and AD model can potentially enhance therapeutic development and further our understanding of AD.

## Methods

### NSC cell line generation

Multiple human induced pluripotent stem cell-derived neural stem cell lines (iPSC-NSCs) were obtained from multiple vendors and tested for basal NSC maintenance and intrinsic neuronal differentiation quality in a small scale (Axol, Millipore, ThermoFisher, MTI global, Tempo Bioscience, Roche). iPSC NSC from MTI Global Stem (HIPTM Neural Stem Cells, BC1 line) and NSC-line2 (gift from Christoph Patsch, Roche (Basel, Switzerland) were chosen based on the following criteria: (1) able to be maintained a homogenous NSC morphology beyond 40 passages; (2) >80% neuronal differentiation efficiency; and (3) fast growth rate at least 1:3 expansion/split ratio. (4) No remaining progenitor cells after differentiation. NSCs were transfected to stably and express inducible ASCL1 and NGN2, transcription factors whose expression has been shown to increase differentiation efficiency in combination with differentiation media (Bardy et al.^28^; Ladewig et al.^30^; Zhang et al.^31^). Transcription factors ASCL1, NGN2, and EGFP were cloned into a vector containing a cumate-inducible promoter (Systembio), then stably transfected using Neon electroporation. Both cell lines were cultured according to the manufacturer’s instructions. Briefly, cells were cultured on flasks coated with a 1:100 Geltrex (ThermoFisher) solution for at least 1 h in a 37 °C cell incubator. Cells were grown in Neural Stem Cell Growth Media and 0.75 μg/ml Puromycin selection marker 37 °C 5% CO_2_ cell culture incubator. Cells were passaged every 3–4 days when confluent using TrypLE Express Enzyme (Gibco) and split at no more than a 1:3 ratio depending on cell density.

### Astrocyte culture

Primary human astrocytes (ThermoFisher) were cultured and passaged according to manufacturer’s instructions in Primary Human Astrocyte Medium on T-650 flasks coated with 1:100 Geltrex (ThermoFisher) solution for at least 1 h in a 37 °C cell incubator. Full volume of media was changed every 3–4 days until cells were confluent. Cells were passaged when confluent using TrypLE Express Enzyme (ThermoFisher) and split at a ratio of up to 1:6.

For astrocyte validation experiments, primary human astrocytes were detached using Accumax (Innovative Cell Technologies) and seeded onto 384-well CellCarrier Ultra imaging plates (PerkinElmer) coated with 1:100 Geltrex (ThermoFisher) solution for at least 1 h in a 37 °C cell incubator. Cells were seeded at a density of 2000 cells/well in Neuron Maintenance Medium. Cells were placed in a 37 °C cell incubator for 24 h to allow for attachment. Aβ42 and antibody treatments were added according to “Aβ42 Oligomer Experiments”. Astrogliosis was validated by immunostaining of the following markers: Guinea Pig anti-GFAP (Synaptic Systems 173004 1:500), Rabbit anti-EAAT1 (Boster PA2185, 1:500), Rabbit anti-vimentin (Cell Signaling 3932S 1:500), Rabbit ALDH1L1 (Abcam ab19029, 1:500).

### NSC differentiation

NAG-NSCs were grown until confluent and then detached using TrypLE Express Enzyme (Gibco) and plated onto a T-650 flask coated with 50 μg/ml poly-D-lysine (Sigma-Aldrich) and 10 μg/ml mouse laminin (ThermoFisher). Cells were plated at a density of 0.7 × 108–1.0 × 10^8^ cells/flasks in Neuron Differentiation Media supplemented 100 μg/ml Cumate (System Biosciences), 1 μM PD0332991 cell cycle inhibitor (Tocris), and 10 μM Y27632 Rock inhibitor (Tocris). Cells were differentiated for 5–7 days with one half volume differentiation media changed every 3 days.

### Neuron replating

Once differentiated, cells were detached using Accumax (Innovative Cell Technologies) supplemented with 5% trehalose dihydrate (Sigma-Aldrich), 1U Papain (Worthington), 10 μM Y27632 (Tocris), 8 mM kynurenic acid (Sigma-Aldrich). Cells were plated using Tecan Fluent automation workstation into 384- or 96-well CellCarrier Ultra imaging plates (PerkinElmer) coated with 50 μg/ml poly-D-lysine (Sigma-Aldrich) and 20 μg/ml recombinant human laminin (Sigma-Aldrich) in Neuron Differentiation Media supplemented with 10 μM Y276342 Rock Inhibitor (Tocris), and 1X RevitaCell (Gibco).

### Astrocyte coculture

Astrocytes were added to neuron culture (5–10 days after neuron replating). Astrocytes were detached using Accumax (Innovative Cell Technologies) and plated using Tecan Fluent automation workstation into 384- or 96-well plates containing differentiated and replated neurons at a density of 4000 or 20,000 cells/well, respectively.

### Neuron–astrocyte coculture automated maintenance

Neurons and astrocytes were cultured in 384- or 96-well Cell Carrier Ultra plates (PerkinElmer) in Neuron Maintenance Medium and half of the volume of culture media was changed using Tecan Fluent automation liquid-handling workstation every 3–4 days for at least 8 weeks and up to 6 months before subsequent experimentation. Tecan Fluent was programmed to utilize its features of automated tip loading, lid removal, and to aspirate half the volume of culture media and add new culture media for up to 30 plates at a time. Barcode-operated plate storage incubator technology was integrated into the system for plate organization and retrieval.

### iPSC microglia culture

iPSC-derived iCell microglia were obtained from multiple vendors and screened for microglia marker expression. Cells from two vendors were used in this publication. iPSC microglia from Cellular Dynamics international were differentiated based on published protocols^65^. BrainXell Inc microglia differentiation was based on published protocol. Briefly, iPSCs were treated with BMP, FGF, and Activin for 2–4 days to induce mesoderm fate, then treated with VEGF and supportive hematopoietic cytokines for 6–10 days to generate hematopoietic progenitors. HPCs were seeded onto Matrigel-coated flasks, treated with IL-34, IDE1 (TGF β1 agonist), and M-CSF for 3–4 weeks to differentiate into microglia. Human iPSC microglia were validated by immunostaining of the following markers: Goat anti-TREM2 (1:500, R&D Systems, AF1828), Mouse anti-MERTK (1:500, Biolegend, 367602), Rabbit anti-IBA1(1:1000, Wako Chem, 019-19741), Rabbit anti-TMEM119 (1:500, Abcam, ab209064), CD33 (1:500, Biolegend, 303302), CX3CR1 (1:500, BioRad, AHP1589), CD64 (1:500, Biolegend, 305012), P2RY12 (1:500, Sigma, HPA014518), CD32 (1:500, Biolegend, 303212), PU.1 (1:500, Cell Signaling, 266S).

Frozen Cells were thawed and immediately seeded at a density of 8000 cells/well of a 384-well plate onto an 8-week-old neuron–astrocyte coculture in Microglia media. BrainPhys neuronal media (Stem Cell Technologies) supplemented with 1X B27 with vitamin A (ThermoFisher), 1X N2 Plus media supplement (R&D Systems), 20 ng/ml BDNF (Peprotech), 20 ng/ml GDNF (Peprotech), 1 mM creatine (Sigma-Aldrich), 200 nM L-ascorbic acid (Sigma-Aldrich), 1 μg/ml mouse laminin (ThermoFisher), 0.5 mM glutamax (ThermoFisher), 0.5X penicillin-streptomycin (ThermoFisher), 1X Normocin (Invivogen), 5 ng/ml TGF-b (Peprotech), 100 ng/ml human IL-34 (Peprotech), 1.5 μg/ml cholesterol (Sigma-Aldrich), 1 ng/ml gondoic acid (Cayman Chemicals), 100 ng/ml oleic acid (Cayman Chemicals), 460 μM Thioglycerol (Sigma-Aldrich), 1X Insulin-Transferrin-Selenium (ThermoFisher), 25 ng/ml rhM-CSF (Peprotech), 5.4 μg/ml Human Insulin Solution (Sigma).

### Monocyte-derived macrophage culture

Monocyte-derived human macrophages were obtained from PromoCell. Macrophages were differentiated into M1 or M2 polarized macrophages by treatment with GM-CSF and M1 Macrophage Generation Medium or M-CSF and M2 Macrophage Generation Medium, respectively (PromoCell). Cells were differentiated for 9 days, then frozen into aliquots. Frozen Cells were thawed and immediately seeded at a density of 8000 cells/well of a 384-well plate onto 8-week-old neuron–astrocyte coculture in Microglia media (above) supplemented with GM-CSF or M-CSF before subsequent experiments.

### Soluble Aβ species generation

AggreSure™ β-amyloid (1–42), Human monomers were purchased from Anaspec and resuspended in DMSO followed by PBS to form a 100 μM solution. Aβ oligomerization was made following a previously published protocol (Stine et al., 2011). Briefly, Aβ42 monomers were subsequently incubated at 4 °C for 24 h, then frozen at −80 °C to stop the oligomerization process. We found that neuronal toxicity is variable between lots, but is consistent within one lot. Five to six lots of Aβ42 monomers were screened at a time and assessed for neurotoxic and the degree of toxicity. The working lot was purchased in bulk (50 mg). During this study in the span of 4 years, 3 rounds of screening were conducted after the bulk working lot of abeta was used up. Total of three lots were purchased in bulk for this study. We noted the variability is improved with GMP versions of Aβ monomer. For fluorescent Aβ42 oligomer experiments, β-amyloid (1–42), HiLyte™ Fluor 555-labeled, Human (Anaspec) was used. For pHrodo experiments, β-amyloid (1–42), Human was labeled with pHrodo™ Green AM Intracellular pH Indicator (Invitrogen) according to manufacturer’s protocol.

### Soluble Aβ species experiments

Prior to experimentation, media volume in wells containing neurons was equalized with liquid-handling automation (Bravo) in order to ensure precise control of concentrations. All Aβ42 oligomers, anti-Aβ, small molecules, inflammatory cytokines were prepared at 10× concentration then added to neuronal culture media at an appropriate volume to ensure the final concentration listed. For the repeated dosing experiments, the media were refreshed 50% at each dosing first, before adding Aβ42 oligomers, and/or anti-Aβ antibody at the specified final concentration.

### Aβ42 oligomer conformation ELISA

To determine the oligomeric species’ of Aβ42 present in sAβ42s, we developed oligomer selective and fibril selective ELISAs. To detect the presence of oligomeric Aβ42, we utilized a 6E10-6E10 assay utilizing the same anti-Aβ42 (6E10) as both capture and detection to selectively bind to oligomeric species containing more than one exposed 6E10 binding site. To further test for oligomeric species, we developed a GT622-6E10 assay using Aβ-oligomer specific antibody (GT622) as capture, and pan-Aβ antibody (6E10) as detection. Finally, we tested for the presence of fibril species using Aβ fibril selective antibody clone (OC) as capture and pan-Aβ antibody (6E10) as detection. sAβ42s to be tested were prepared according to “Aβ42 Oligomer Generation” protocol. Clear, flat bottom immuno nonsterile maxisorp 384-well (Nunc) were coated with 100 ng/ml of different anti-Aβ42 antibody (6E10, Biolegend, 803003; GTX622, Genetex, GTX635160; OC, Millipore, AB2286) in 0.05 M sodium carbonate buffer, pH 9.6 overnight. Plates were washed 3× with 0.05% Tween-20 in 1X PBS, then blocked in 0.5% BSA + 15PPM Proclin in 1X PBS, pH 7.4 for 1 h. All samples were quantified against Aβ42 monomer diluted to 1 μg/ml in 0.5% BSA + 0.05% Tween-20 + 0.35 M NaCl + 0.25% CHAPS + 5 mM EDTA in 1X PBS, pH 7.4 (Assay Buffer), then diluted twofold to 15.625 ng/ml. Sample Aβ42 oligomers were diluted to 1 μg/ml in Assay Buffer, then diluted threefold to 37 nM. Blocked plate was then washed 3× in 0.05% Tween-20 in 1X PBS, then samples, standards, and controls were added and incubated at 4 °C overnight. After sample incubation, plate was washed 6× with 0.05% Tween-20 in 1X PBS, then 100 ng/ml conjugate antibody in Assay Buffer (6E10, Biologend, 803003) was added and incubated for 1 h at RT. After incubation, plate was washed 6× with 0.05% Tween-20 in 1X PBS, then Strep-Avidin Poly 80 HRP detection antibody was added at a dilution of 1:10,000 in Assay Buffer and incubated for 45 min at RT. After incubation, plate was washed 6× with 0.05% Tween-20 in 1X PBS, and TMB substrate was added to each well, then incubated for 10–15 min. After appropriate color was developed, 1 M H_3_PO_4_ was added to quench the reaction. Finally, plate O.D. was read at 450–630 nm.

### APOE genotyping

Neuronal cells to be genotyped were harvested and lysed according to “Mammalian Cells Lysate” instructions included in PureLink™ Genomic DNA Mini Kit (Invitrogen). Genomic DNA was purified according to manufacturer instructions included in PureLink™ Genomic DNA Mini Kit (Invitrogen). DNA was quantified using Nanodrop spectrophotometer, then diluted to 15 ng/µL.

APOE genotyping PCR was performed using EzWay Direct ApoE Genotyping Kit (Koma Biotech), which includes a proprietary ApoE primer mixture for E2 (Cys112/Cys158), E3 (Cys112/Arg158), and E4 (Arg112/Arg158). Fifteen nanograms of purified DNA for each sample was added to a 25 µL sample reaction. Primers PCR conditions and cycling was performed as follows:

Initial denaturation at 95 °C for 15 min.

Denaturation at 95 °C for 30 s (35 cycles).

Annealing at 65 °C for 30 s (35 cycles).

Extension at 72 °C for 1 min (35 cycles).

Final extension at 72 °C for 10 min.

Amplified DNA was run on a 1% agarose gel at 100 V for 1 h, then imaged.

The genotype of iPSC-NSC neurons from MTI Global Stem is ApoE4/4, while the NSC-line2 is ApoE3/3. We observed similar phenotypes in both of these cell lines, and thus ApoE genotype status does not appear to affect the phenotypes reported throughout this study. Human primary astrocytes (ThermoFisher) and iPSC microglia (CDI and BrainXell) are ApoE 3/3 (Table 1).

**Table 1:** ApoE genotype status.

Cell line	Vendor	ApoE genotype
BC1 NAG NSC (MTI Global Stem)	MTI Global Stem	E4/E4
NSC-line2 (Christoph Patsche)	Roche	E3/E3
Primary human astrocytes	ThermoFisher	E3/E3
iCell Microglia 01279	Fujifilm CDI	E3/E3
iPSC Microglia	BrainXell	E3/E3

### Immunofluorescence staining

Cells were fixed with 4% PFA, 4% sucrose at room temperature for 20 min using Bravo automation. Fixed cells were then washed two times with PBS using Biotek 406 microplate washer (Beckman Coulter), followed by permeabilization and blocking by incubation with a solution containing 1X PBS, 0.1% Triton X-100, 2% donkey serum, and 1% BSA at room temperature for 30 min. Blocking solution was removed and cells were incubated with primary antibodies in blocking solution overnight at 4 °C. After washing six times with PBS on Biotek 406 cell plate washer (Beckman Coulter), cells were then incubated with fluorophore-conjugated secondary antibodies (Jackson Immuno Research Laboratories, Inc.) for 1 h at room temperature in the dark to avoid photobleaching. Cells were then washed six more times with PBS before imaging. Fluorescent images were captured using an InCell6000 confocal microscope (GE Healthcare Life Sciences). The image analysis was performed with InCell 6000 analysis software.

### Small-molecule focused screen and IC50 curve follow-up

A focused library of 70 small molecules and natural products (Genentech, Selleckchem, Tocris Biosciences) implicated with neuroprotective properties were tested using a cell rescue assay in a 384-well microplate format (Cell Carrier Ultra). For double culture screen, mature human neuronal cultures (12 weeks+) were equalized in volume immediately before experiments using Bravo Automated liquid-handling platform (Agilent) to account for evaporative edge effects. For the triple culture screen, mature human neuron cultures (12 weeks+) were equalized in volume using Bravo Automated liquid-handling platform (Agilent), then microglia were plated according to the “iPSC Microglia Culture” section. After 3 days and immediately before experiments, triple culture volumes were equalized using Bravo Automated liquid-handling platform (Agilent) to account for evaporative edge effects. For both double and triple culture screens, pharmacological treatments were prepared from 10 mM DMSO stocks. 10 mM drug stocks were first diluted into Neuron Maintenance media to create intermediate stock solutions ranging from 1 to 1000 μM. Double screen drug concentrations used: 50, 25, 12.5, 6.25 μM. Triple screen concentrations used: 50, 12.5, 3.125, and 0.78 μM. Triple screen concentration was reduced to lower drug toxicity and increase hit rate. Intermediate stocks were then diluted to create 10X concentrations of drugs, which were serially diluted to generate a range of concentrations necessary to generate a dose–response profile. Final DMSO in treatments was kept at below 0.1%. Abeta oligomers were added to wells ~1 h after drug treatment to a final concentration of 5 μM Abeta oligomer per well. Cells were incubated with compounds and Abeta for 3–7 days (depending on Abeta lot strength and pharmacological profile of drugs tested) at 37 °C and 5% CO_2_. Cells were fixed with 4% Paraformaldehyde solution (Electron Microscopy Sciences), 4% sucrose solution. Fixed cells were stained for various neuronal markers for dendrites (Map2), synapse (Synapsin1/2), nucleus (CUX2), and axons (Tuj1). Plates were imaged using the InCell6000 confocal microscope (GE) and then analyzed with the InCell 6000 image analysis software.

### Immunostaining antibodies

The following primary antibodies were used for immunostaining experiments: Chicken anti-MAP2 (1:2000, GeneTex, GTX85455), Guinea pig anti-Synapsin 1/2 (1:750, Synaptic Systems, #106 004), Mouse anti-Tau HT7 (1:500, Invitrogen, MN1000), Mouse anti-Aβ 6E10 (1:500, Biolegend, #803003), Rabbit anti-CUX2 (1:500, Abcam, ab140329), Rat anti-CTIP2 (1:500, Abcam, ab19465), Guinea pig anti-vGlut2 (1:1000, Synaptic systems, #135304), Mouse anti-Shank (1:200, Millipore N23B/49), Mouse anti-PSD95 (1:200, Millipore, K28/43), Mouse anti-GluR1 (1:200, Millipore), Mouse anti-PanShank (1:200, Millipore, N23B/49), Mouse anti-GluR2 (1:200, Millipore), Mouse anti-PanSAPAP (1:200, Millipore), Mouse anti-NR1 (1:200, Millipore), Rabbit anti-phospho-Tau S396-404 (1:3000, Genentech), Rabbit anti-phospho-Tau S235 (1:1000, ThermoFisher), Mouse anti-β-Tubulin Tuj1 (1:500, Biolegend, #801202), Chick anti-Neurofilament-Heavy Chain (1:3000, Abcam), Rabbit anti-Iba1 (1:1000, Wako Chem, 019-19741), Rabbit anti-TMEM119 (1:500, Abcam, ab209064). Rabbit anti-ApoE (1:500, ThermoFisher 16H22L18), Rabbit anti-APP (1:500, Abcam ab32136), Guinea pig anti-GFAP (1:500 Synaptic System 173004), Rabbti anti-ALDH1L1 (1:500, Abcam ab190298), Rabbit anti-Vimentin (1:500, Cell Signaling 3932S), Rabbit anti-EAAT1 (1:500, Boster PA2185).

Secondary antibodies were obtained from Jackson ImmunoResearch. Whole antibodies made in a donkey host with IgG (H + L) specificity and minimal cross-reactivity were used. For immunostaining experiments, secondary antibodies against their appropriate primary antibody species and conjugated to either DyLight405, AlexaFluor488, Cyanine Cy3, or AlexaFluor647. All 405- and 488-conjugated antibodies were used at 1:200, all cy3 conjugated antibodies were used at 1:600, and all 647-conjugated antibodies were used at 1:800.

### Image analysis

Image analysis was performed using supplied InCell6000 analysis software, IN Cell Software was utilized to segment cellular regions of interest such as dendrites, cell bodies, and axons. During image acquisition, 9 images are collected per well, which are then summed into a value for each well total of 4 wells. Percent rescue and phospho-Tau induction were calculated as follows:

1 $${{{{{\rm{MAP2}}}}}}\,{{{{{\rm{Percent}}}}}}\,{{{{{\rm{rescue}}}}}}=\frac{\big({{Area}}_{{{experimental}}\,{{condition}}}-({{mean}}({{Area}}_{A\beta 42{{treated}}}))}{{{mean}}({{Area}}_{{{{{{\rm{untreated}}}}}}})-{{mean}}({{Area}}_{A\beta 42{{treated}}})}$$

2 $${{{{{\rm{Synapsin}}}}}}\,{{{{{\rm{count}}}}}}\,{{{{{\rm{rescue}}}}}}=\frac{\big({{Count}}_{{{experimental}}\,{{condition}}}-({{mean}}({{Count}}_{A\beta 42{{treated}}}))}{{{mean}}({{Count}}{\,}_{{{untreated}}})-{{mean}}({{Count}}_{A\beta 42{{treated}}})}$$

3 $${{{{{\rm{p-Tau}}}}}}\,{{{{{\rm{induction}}}}}}=\\ \frac{{(D{\times}A({{pTau}}396-404))}_{{{experimental}}\,{{condition}}}/(D{\times}A{({{total}}\,{{Tau}})}_{{{experimental}}\,{{condition}}})\big)}{{(D{\times}A({{pTau}}396-404))}_{{{untreated}}}/(D{\times}A{({{total}}\,{{Tau}})}_{{{untreated}}})}$$

Replicates of 4 were averaged and plotted with error bars representing SEM. *D* × *A* = total integrated intensity. IC50 curves were fitted utilizing Prism software.

### Peggy Sue automated western

Cells were lysed with Pierce IP lysis buffer (ThermoFisher #87787) containing protease and phosphatase inhibitor cocktail and cleared by centrifugation. Lysates concentration was quantified using the Micro BCA Protein Assay Kit (ThermoFisher #23235) and adjusted with lysis buffer. Sample lysates were then combined with a 5X master mix (ProteinSimple) to achieve a final concentration of 1X sample buffer, in the presence of fluorescent standards and 40 mM dithiothreitol (DTT). The mixture was denatured at 95 °C for 5 min, and proteins were separated using the Peggy Sue system (ProteinSimple, San Jose, California, USA) with a 12–230 kDa Separation Module (ProteinSimple #SM-S001), according to the manufacturer’s standard protocol. Phosphorylated cJun was immune-probed with anti-phospho-cJun (Cell Signaling, #2361), while the secondary antibody was from the ProteinSimple kit. Both antibodies were diluted using an antibody diluent (ProteinSimple). GAPDH was used as an internal reference. The digital image was analyzed with Compass for Simple Western software.

### Tau fraction western

#### Sample preparation and fractionation

iPSC-derived neurons cultured in 96-well plates for 3 months were treated with sAβ42s as in the Aβ42 Oligomer Experiments section above. Cells were treated twice per week at media change, after three weeks the cells were lysed in 50 µl/well NP40 buffer (Thermo FNN0021) with protease and phosphatase inhibitors. Three to four wells were pooled as one sample and fractionated. Lysate was spun at 20k × *g* for 30 min at 4 °C. The supernatant was taken as the soluble fraction. The resulting pellet was resuspended vigorously in RIPA (Thermo 89900) with 1% sarkosyl detergent (Sigma 61743) and protease and phosphatase inhibitors by pipetting through a small aperture until a homogeneous solution was obtained. The solution was spun at 20k × *g* for 30 min at 4 °C. The supernatant was removed and the remaining sarkosyl insoluble pellet was resuspended in sample buffer 4x LDS (Genscript M00676) with 5% beta-mercaptoethanol before electrophoresis. The soluble fraction was assayed by Micro BCA Protein Assay Kit (ThermoFisher #23235) to determine protein concentration.

### Immunoblotting

Equal soluble protein (2 μg/lane) and a proportional amount of the corresponding insoluble sample were loaded into a 4–12% Bis-Tris Gel (Invitrogen WG1402BOX) and separated in MES buffer (Invitrogen NP0002). Protein was blotted by the iBlot2 device and compatible PVDF stacks (Invitrogen IB24001). Membranes were fixed in 4% PFA (Electron Microscopy Sciences) in PBS for 15 min at RT. Membranes were blocked in blocking buffer 10% donkey serum and 5% BSA (Jackson Immunoresearch) in PBS for 1 h RT. Primary antibodies were diluted in blocking buffer 1:1000 and incubated overnight at 4 °C, rocking (Tau HT7 Thermo MN1000, Histone H3 4499S Cell Signaling Technology, 3R Tau 05-803 EMDMillipore, 4R Tau 05-804 EMDMillipore). Secondary antibody (peroxidase-Donkey anti-mouse 715-035-150, peroxidase-Donkey anti-rabbit 711-033-152, Jackson Immunoresearch) was diluted in blocking buffer (1:20,000) and incubated for 1 h RT rocking. Bands were detected by ECL with Supersignal Femto (Thermo 34094) on AzureBiosystems Azure500 chemi detection.

### Gyros Aβ42 immunoassay

The supernatant was collected at the end of each experiment using Bravo liquid handler technology prior to cell fixation and used immediately or frozen at −80 °C. Sample supernatants were diluted 1:500 using 0.05% Tween-20 in 1X PBS. Capture antibody Biotinylated mouse anti-β-amyloid antibody 1–16 6E10 (Biolegend) was diluted to a working concentration of 100 μg/ml in 0.05% Tween-20 in 1X PBS. Detection antibody AlexaFluor647 labeled (ThermoFisher, AlexaFluor647 Antibody Labeling Kit) rabbit anti-Beta Amyloid Monoclonal Antibody (ThermoFisher) was diluted to a working concentration of 25 nM in Rexxip F buffer (Gyros Protein Technologies). All samples were quantified against AggreSure Beta-Amyloid (1–42), Human (Anaspec) oligomerized according to Aβ42 Oligomer Generation protocol above. The standard reagent was diluted to 50 nM in 0.05% Tween-20 in 1X PBS and diluted twofold to 3 pM. Capture and detection antibodies, standards, and samples were loaded onto fully-skirted, 96-well polypropylene plates (ThermoFisher) and run on Gyrolab xPand system on a 1000 nL CD. Assay ran according to Gyros manufacturer standard protocol utilized a 3-step ELISA assay system protocol and two wash buffers: 0.05% Tween-20 in 1X PBS wash buffer and pH 11 wash buffer (Gyros Protein Technologies). Gyros version 6.4 software (Gyros Protein Technologies) was used to measure protein concentrations from Gyros immunoassays.

### Incucyte time-lapse movie acquisition

Incucyte movies were acquired according to the manufacturer’s instructions. Images were programmed to acquire once every 30 min in the brightfield spectrum, green fluorescence channel (400 ms acquisition time), and red fluorescence channel (800 ms acquisition time). One image per well in a 384-well plate with a ×20 objective.

### Neural stem cell growth medium

0.5X DMEM/F12 (ThermoFisher), 0.5X Neurobasal (ThermoFisher), 1X B27 no Vitamin A (ThermoFisher), 1X N2 (ThermoFisher), 20 ng/ml BDNF (Peprotech), 20 ng/ml FGF-basic (Peprotech), 20 ng/ml EGF (Peprotech), 0.5 mM Glutamax (Gibco), 0.11 mM β-Mercaptoethanol (Sigma-Aldrich), 1X Normocin (InvivoGen), 50 U/ml Penicillin-Streptomycin (ThermoFisher).

### Astrocyte medium

1X DMEM/F12 (ThermoFisher), 1X N2 (ThermoFisher), 10% FBS (ThermoFisher), 1X Normocin (InvivoGen), 50 U/ml Penicillin-Streptomycin (ThermoFisher).

### Neuron differentiation medium

0.5X DMEM/F12 (ThermoFisher), 0.5X Neurobasal (ThermoFisher), 1X B27 with Vitamin A (ThermoFisher), 1X N2 (ThermoFisher), 5 μg/ml Cholesterol (Sigma-Aldrich), 1 mM Creatine (Sigma-Aldrich), 100 μM Ascorbic Acid, 0.5 mM cAMP (Sigma-Aldrich), 20 ng/ml BDNF (Peprotech), 20 ng/ml GDNF (Peprotech), 1 μg/ml Laminin, 0.5 mM Glutamax (ThermoFisher), 1X Normocin (InvivoGen), 50 U/ml Penicillin-Streptomycin (ThermoFisher).

### Neuron maintenance medium

1X BrainPhys Basal (StemCell Technology), 1X B27 with Vitamin A (ThermoFisher), 1X N2 (ThermoFisher), 5 μg/ml Cholesterol (Sigma-Aldrich), 1 mM Creatine (Sigma-Aldrich), 10 nM β-estradiol, 200 nM Ascorbic Acid, 1 mM cAMP (Sigma-Aldrich), 20 ng/ml BDNF (Peprotech), 20 ng/ml GDNF (Peprotech), 1 μg/ml Laminin, 0.5 mM Glutamax (ThermoFisher), 1 ng/ml TGF-β1 (Peprotech), 1X Normocin (InvivoGen), 50 U/ml Penicillin-Streptomycin (ThermoFisher).

### Microglia media

BrainPhys neuronal media (Stem Cell Technologies) supplemented with 1X B27 with vitamin A (ThermoFisher), 1X N2 Plus media supplement (R&D Systems), 20 ng/ml BDNF (Peprotech), 20 ng/ml GDNF (Peprotech), 1 mM creatine (Sigma-Aldrich), 200 nM L-ascorbic acid (Sigma-Aldrich), 1 μg/ml mouse laminin (ThermoFisher), 0.5 mM glutamax (ThermoFisher), 0.5X penicillin-streptomycin (ThermoFisher), 1X Normocin (Invivogen), 5 ng/ml TGF-b (Peprotech), 100 ng/ml human IL-34 (Peprotech), 1.5 μg/ml cholesterol (Sigma-Aldrich), 1 ng/ml gondoic acid (Cayman Chemicals), 100 ng/ml oleic acid (Cayman Chemicals), 460 μM Thioglycerol (Sigma-Aldrich), 1X Insulin-Transferrin-Selenium (ThermoFisher), 25 ng/ml rhM-CSF (Peprotech), and 5.4 μg/ml Human Insulin Solution (Sigma).

### Statistical analysis summary

Image analysis was performed using supplied InCell6000 analysis software, InCell Investigator. The software was utilized to segment cellular regions of interest such as dendrites, cell bodies, and axons. During image acquisition, 9 images are collected per well, which are then summed into a value for each well total of 4 wells. Percent rescue and phospho-Tau induction were calculated as follows:

$${{{{{\rm{MAP2}}}}}}\,{{{{{\rm{Percent}}}}}}\,{{{{{\rm{rescue}}}}}}=\frac{\big({{Area}}_{{{experimental}}\,{{condition}}}-({{mean}}({{Area}}_{A\beta 42{{treated}}}))}{{{mean}}({{Area}}{\,}_{{{untreated}}})-{{mean}}({{Area}}_{A\beta 42{{treated}}})}\quad(1)$$

$${{{{{\rm{Synapsin}}}}}}\,{{{{{\rm{count}}}}}}\,{{{{{\rm{rescue}}}}}}=\frac{\big({{Count}}_{{{experimental}}\,{{condition}}}-({{mean}}({{Count}}_{A\beta 42{{treated}}}))}{{{mean}}({{Count}}{\,}_{{{untreated}}})-{{mean}}({{Count}}_{A\beta 42{{treated}}})}\quad(2)$$

$${{{{{\rm{p-Tau}}}}}}\,{{{{{\rm{induction}}}}}}=\\ \frac{{(D{\times}A({{pTau}}396-404))}_{{{experimental}}\,{{condition}}}/(D{\times}A{({{total}}\,{{Tau}})}_{{{experimental}}\,{{condition}}})\big)}{{(D{\times}A({{pTau}}396-404))}_{{{untreated}}}/(D{\times}A{({{total}}\,{{Tau}})}_{{{untreated}}})}\quad(3)$$

4 $$Z\,{{{{{\rm{factor}}}}}}=1-\frac{3(({{Stdev}}_{{{noAbeta}}})+({{Stdev}}_{A\beta 42{{treated}}}))}{({{Mean}}{\,}_{{{noAbeta}}})-({{Mean}}_{A\beta 42{{treated}}})}$$

*D* × *A* = total integrated intensity. All statistical analyses were performed in Prism software using a two-way ANOVA followed by program-recommended post hoc test for each experiment. Each experiment has *N* at least four wells; each well with 1000+ neurons as a sum or average of one well quantified. Data in graphs are expressed as mean values ± s.e.m. Error bars represent s.e.m.

### Statistics and reproducibility

All graphs shown are taken from a representative experiment. All experiments shown were repeated independently at least three times with similar results.

### Reporting summary

Further information on research design is available in the Nature Research Reporting Summary linked to this article.

## Supplementary information

Supplementary Information

Description of Additional Supplementary Files

Supplementary Movie 1

Supplementary Movie 2

Supplementary Movie 3

Reporting summary

## Data Availability

Full scans of the gels and blots are available in Source data file. The screen results generated in this study are provided in the Supplementary Information file. All raw data used to make all quantitative graphs are included in the Supplementary Information file. All other relevant data are available from the corresponding author upon reasonable request. All processed data supporting the findings of this study are available within the article and its Supplementary Information files. A reporting summary for this article is available as a Supplementary Information file. Source data are provided with this paper.

## Source data

Source Data
