# Supplementary material for: Improved modeling of human AD with an automated culturing platform for iPSC neurons, astrocytes and microglia
Source: Nat Commun. 2021 Sep 1;12:5220. doi: 10.1038/s41467-021-25344-6 (PMC8410795; doi:10.1038/s41467-021-25344-6)
Supplement: Supplementary file 1 — Supplementary Information [file 41467_2021_25344_MOESM1_ESM.pdf]

Supplementary Fig. 1

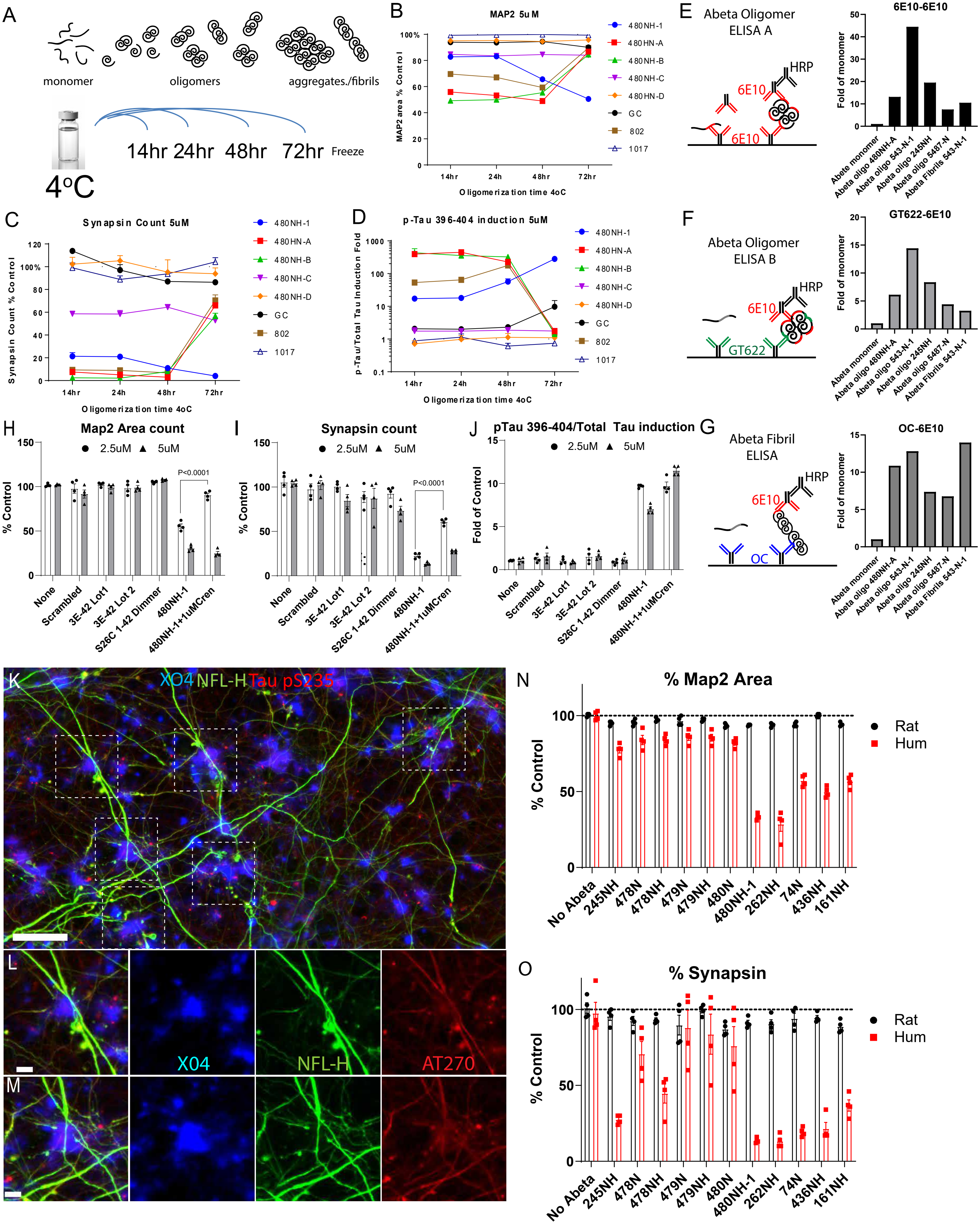

Supplementary Fig. 1 | sAβ42s are selectively toxic to human neurons. (A) sAβ42s were generated by resuspending lyophilized Aβ42 monomers in PBS and incubating monomers at 4C for 14, 24, 48, 72 hours then frozen. **(B-D)** Several lots of Aβ42 monomers oligomerized for 14, 24, 48, and 72 hours were assessed for dendrite toxicity (MAP2) **(B)**, synapse loss (Synapsin 1/2) **(C)**, and p-Tau induction (S396/S404) **(D)**. **(E-G)** Several lots of sAβ42s oligomerized for 24 hours were characterized for oligomeric and fibril conformation using Aβ oligomer selective and Aβ fibril selective ELISA assays. 6E10-6E10 assay utilizing the same anti-Aβ42 (6E10) for capture and detection to selectively bind to oligomeric Aβ42 species **(E),** GT622-6E10 oligomer assay uses Aβ oligomer specificity antibody clone GT622 as capture and pan Aβ antibody clone (6E10) as detection **(F)**, OC-6E10 assay uses Aβ fibril selective antibody clone OC as capture and pan Aβ antibody clone (6E10) as detection. All values were normalized to Aβ monomer negative control, and Aβ42 fibrils were generated by oligomerization in 37°C as a positive control to demonstrate the specificity of this assay. **(H-J)** Several lots of Aβ42 monomers and scramble control were tested at 0, 2.5, 5 μM for dose-response toxicity in dendrites (MAP2) **(H)**, synapse loss (Synapsin 1/2) **(I)**, and p-Tau induction (S396/S404) **(J)**. **(K)** Rat cortical neurons treated with 5μM sAβ42s for 7 days form many plaque-like, Methoxy-X04 positive structures (blue). A few of these plaque-like structures are surrounded by dystrophic neurite-like blebbings of NFL-H (green), and phospho-Tau (AT270, red). Neuritic plaques are indicated by dotted white boxes. **(L-M)** Zoomed in images of B showing axonal swelling (NFL-H; green) and p-Tau induction (S235; red) in axons around Aβ-plaque structures (Methoxy-X04; blue). Concurrently, **(N-O)** rat neurons fail to show Aβ42 oligomer toxicity in response to many lots of Aβ42 oligomer preparations in comparison to human neurons in terms of the dendrite (MAP2) loss **(N)** and severe synapse loss (Synapsin 1/2) **(O)**. Data are presented as mean values +/- SEM and *n*=4 wells. **(B-D, H-J, N-O)**. Scale bars = 100μm **(K)**, 20μm **(L, M)**.

Supplementary Fig. 2

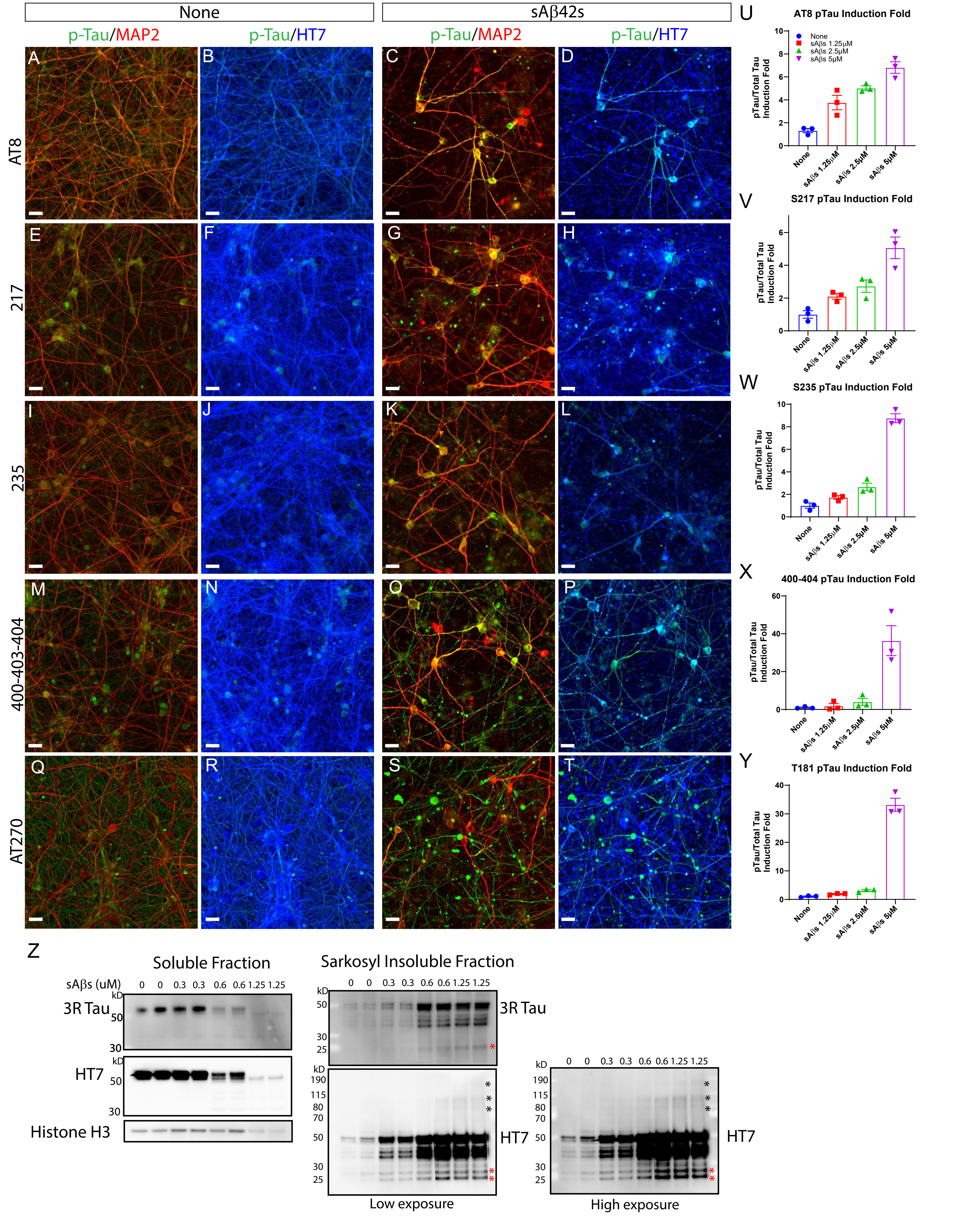

Supplementary Fig. 2 | Additional characterization of Tau pathologies. (A-D) sAβ42s treatment at 5 μM induces somatodendritic accumulation of Tau (overlap with MAP2, third panel) and phosphorylation at S202/T205 and as detected by AT8 antibody (green). **(E-H)** Staining of Tau phosphorylation site S217, **(I-L)** S235, **(M-P)** S400/T403/S404, and **(Q-T)** T181 (AT270) **(U-Y)** Quantification of induction of phosphorylated Tau increases in dose-response to sAβ42s treatment concentration; induction fold calculated by the ratio of p-Tau area to total Tau (HT7) area in $A\beta_{treated}$ induction over the ratio of p-Tau area to total Tau (HT7) in the untreated control. Data are presented as mean values +/- SEM and *n*=4 wells. Scale bars 50 μm. **(Z)** Western blot images showing soluble (right) and insoluble (left) fractions of protein lysates obtained from iPSC neurons and astrocytes treated with 0, 0.3, 0.6, or 1.25μM sAβ42s twice weekly for three weeks, then probed for 3R Tau protein, total Tau (HT7) and loading control histone H3. Upon treatment with soluble Aβ species, there is a dose-dependent increase in the insoluble 3R and total Tau and depletion of these proteins from the soluble fraction. In high concentrations of soluble Aβ species, there are lower molecular weight truncated Tau proteins (red asterisks) and larger molecular weight Tau aggregates (black asterisks).

Supplementary Fig. 3

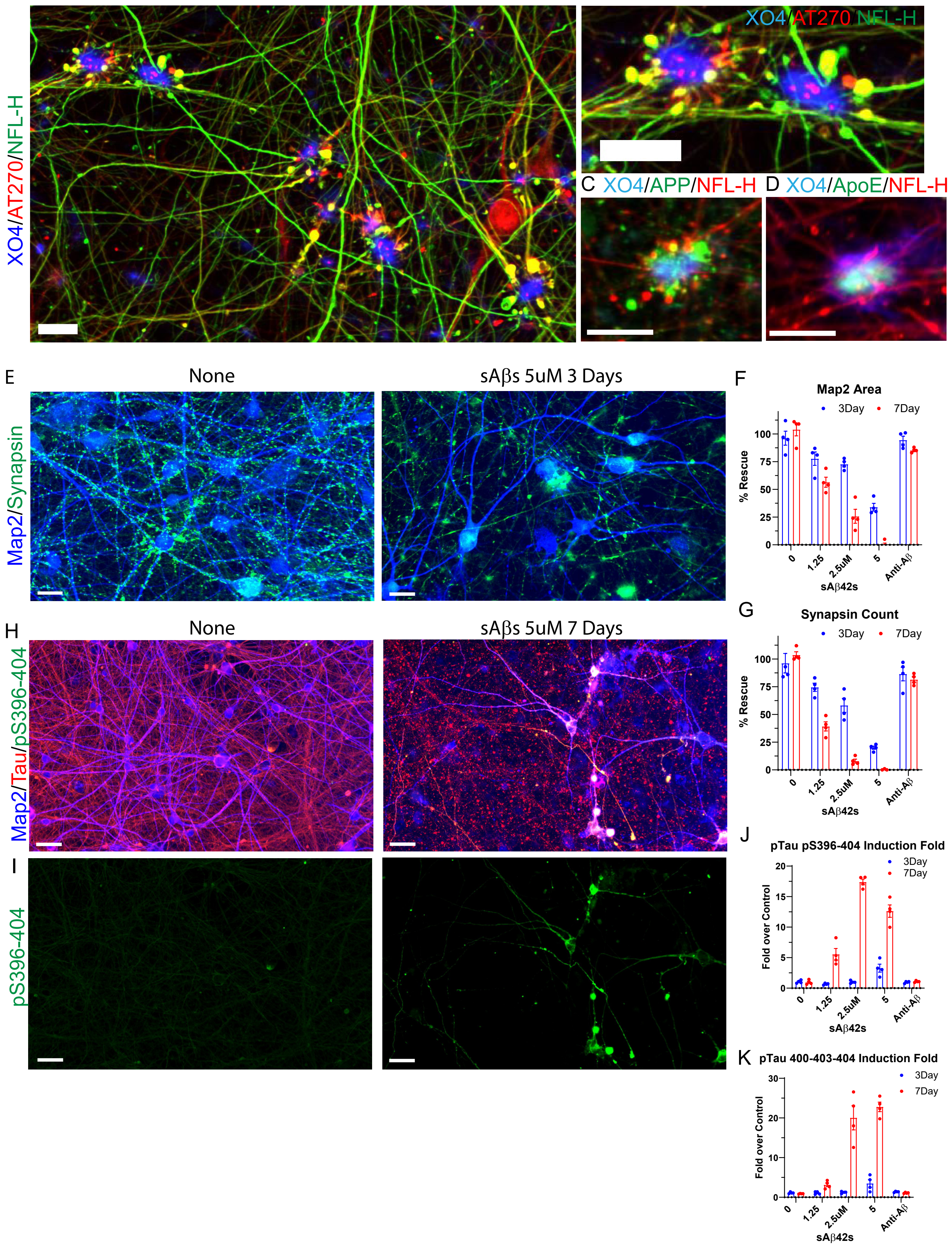

Supplementary Fig. 3 | NSC-NAG Line 2 has similar sAβ42s-induced phenotypes. (A) NAG-NSC Line 2 and primary astrocytes treated with 5 μM sAβ42s for 7 days and stained for Aβ-plaque structures (Methoxy-X04; blue), axons (NFL-H; green), and p-Tau (AT270; red). Right, the zoomed in image showing neuritic plaque. Scale bar = 50um. **(B)** NAG-NSC Line 2 and primary astrocytes treated with 5μM soluble Aβ species for 7 days show loss of dendrites (MAP2, blue) and loss of synapses (synapsin, green) compared to no treatment on right. Scale bar = 50um **(C)** NAG-NSC Line 2 and primary astrocytes treated repeatedly treated with 1.25 μM sAβ42s 3 times and stained with additional neuritic plaque markers APP (green), XO4 (Methoxy-X04; blue), and axons (NFL-H; green). Scale bar = 50um **(D)** Triple culture of NAG-NSC Line2, astrocytes, and iPSC microglia repeatedly treated with 1.25 μM sAβ42s for 3 times and stained with additional plaque markers ApoE (D, green), XO4 (Methoxy-X04; blue), and axons (NFL-H; green). Scale bar = 50um **(E-G)** Quantification of MAP2 and synapsin demonstrate dose-dependent and time-dependent loss of dendrites (MAP2) and synapses (synapsin), and both can be rescued with treatment with anti-Aβ antibody. Scale bar = 100 um. **(H-K)** NAG-NSC Line 2 and primary astrocytes treated with 5uM sAβ42s for 7 days show loss of dendrites (MAP2, blue), Tau fragmentation (HT7, red), as well as upregulation and mislocalization of phospho-Tau (pS396-404, green) from axons to cell bodies and dendrites **(I). (J-K)** Quantification of phospho-Tau p396-404) **(J)** and phospho-Tau p400-403-404 **(K)** fold induction to show that phospho-Tau is upregulated in a dose and time-dependent manner, and this can be blocked with the treatment of anti-Aβ antibody. Data are presented as mean values +/- SEM and *n*=4 wells.

Supplementary Fig. 4

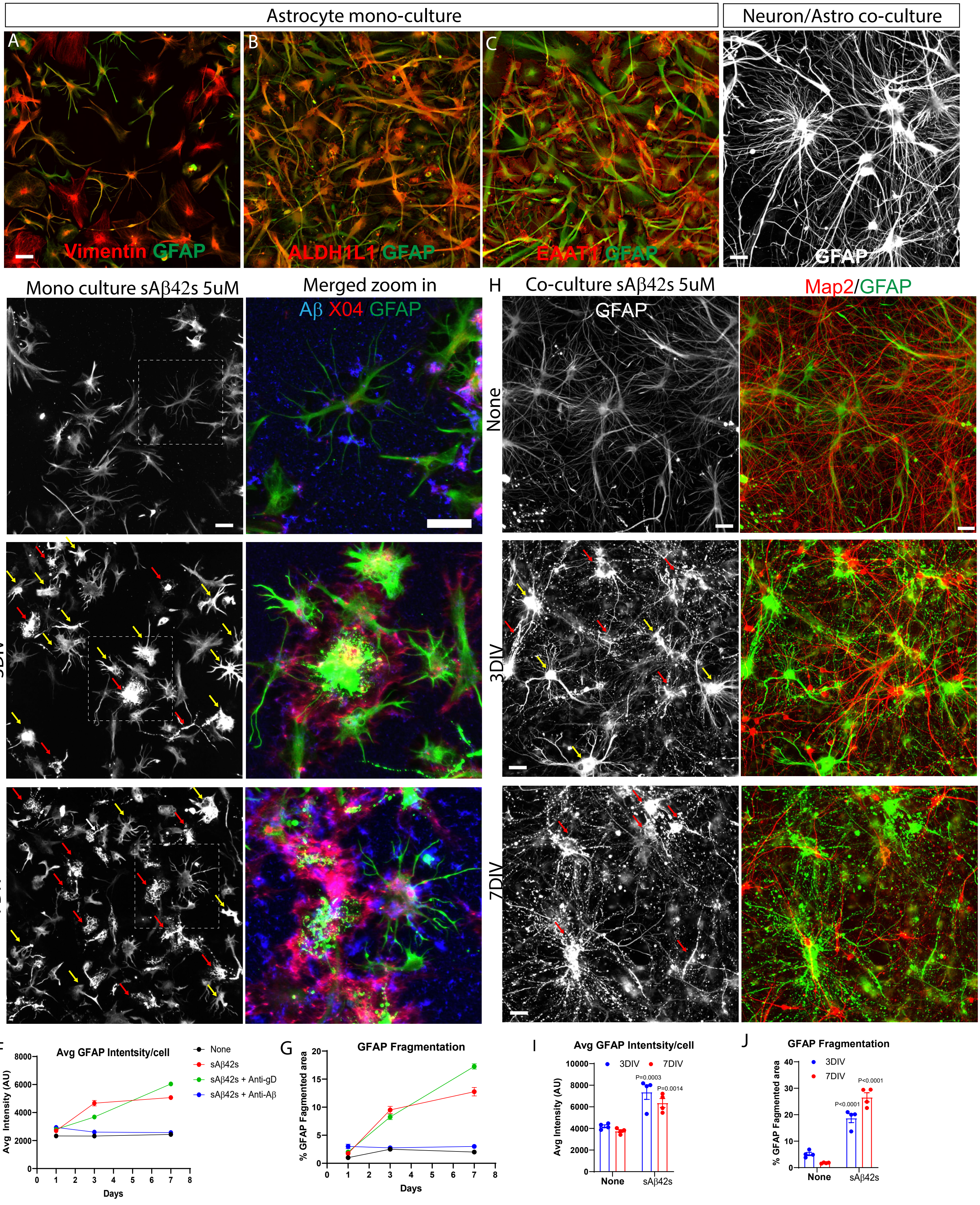

Supplementary Fig. 4 | sAβ42s upregulate GFAP expression and induce GFAP fragmentation (A-C) Primary human astrocytes cultured alone in Neuron Maintenance Medium express astrocyte markers GFAP (green), Vimentin (red, A), ALDH1L1 (red, B), and EAAT1 (red, C). **(D)** Primary human astrocytes cocultured with neurons in Neuron Maintenance Medium develop elaborate processes and more mature morphology (GFAP, white). **(E)** Primary human astrocytes cultured alone in Neuron Maintenance Medium upregulated GFAP (right, white; left, green) starting at 3DIV upon treatment with 5μM sAβ42s, aggregate Aβ (6E10, blue), and form diffuse dye-positive structures (Methoxy-X04, red) that are morphologically different from dye-positive structures that microglia form. At 1DIV (top), we observe small aggregates of Aβ around cell processes that grow and begin to result in some cell death, which worsens at 7DIV. Yellow arrows indicate astrocytes with increased GFAP expression. Red arrows indicate dead/dying cells. The white dotted box indicates the zoomed in regions on the right. **(F)** Quantification of average GFAP intensity/cell shows that at 3DIV astrocytes treated with soluble Aβ species upregulate GFAP, and this is blocked by treatment with anti-Aβ antibody. **(G)** Cell death quantified by fragmentation of the cell body using GFAP shows that primary human astrocytes treated with sAβ42s show marked cell death at 3DIV which worsens at 7DIV. **(H-J)** Primary human astrocytes cocultured with neurons treated with 5μM sAβ42s also demonstrate similar upregulation of GFAP **(I)** and cell fragmentation indicating cell death **(J)** in a dose- and time-dependent manner**.** Data are presented as mean values +/- SEM and *n*=4 wells. Two-way ANOVA with Sidak's multiple comparisons test. Scale bar=100um.

Supplementary Fig. 5

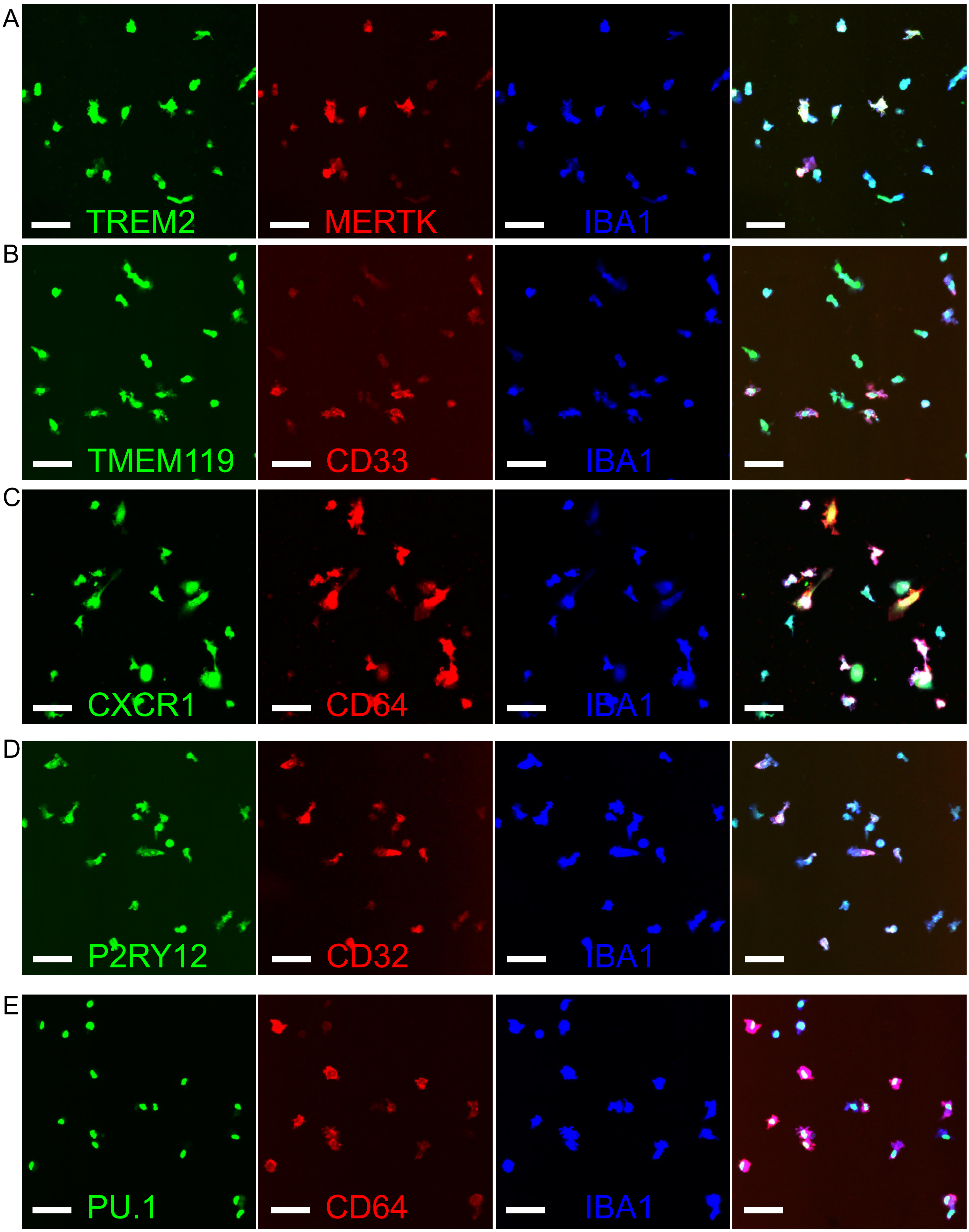

Supplementary Fig. 5 | Human iPSC microglia express common microglial markers and have typical ramified morphology. (A-E) iPSC derived microglia were stained with antibodies against microglia markers: TREM2, TMEM119, CXCR1, P2RY12, PU.1 (green); MERTK, CD33, CD64, CD32 (red); IBA1 (blue). Scale bar = 50μm.

Supplementary Fig. 6

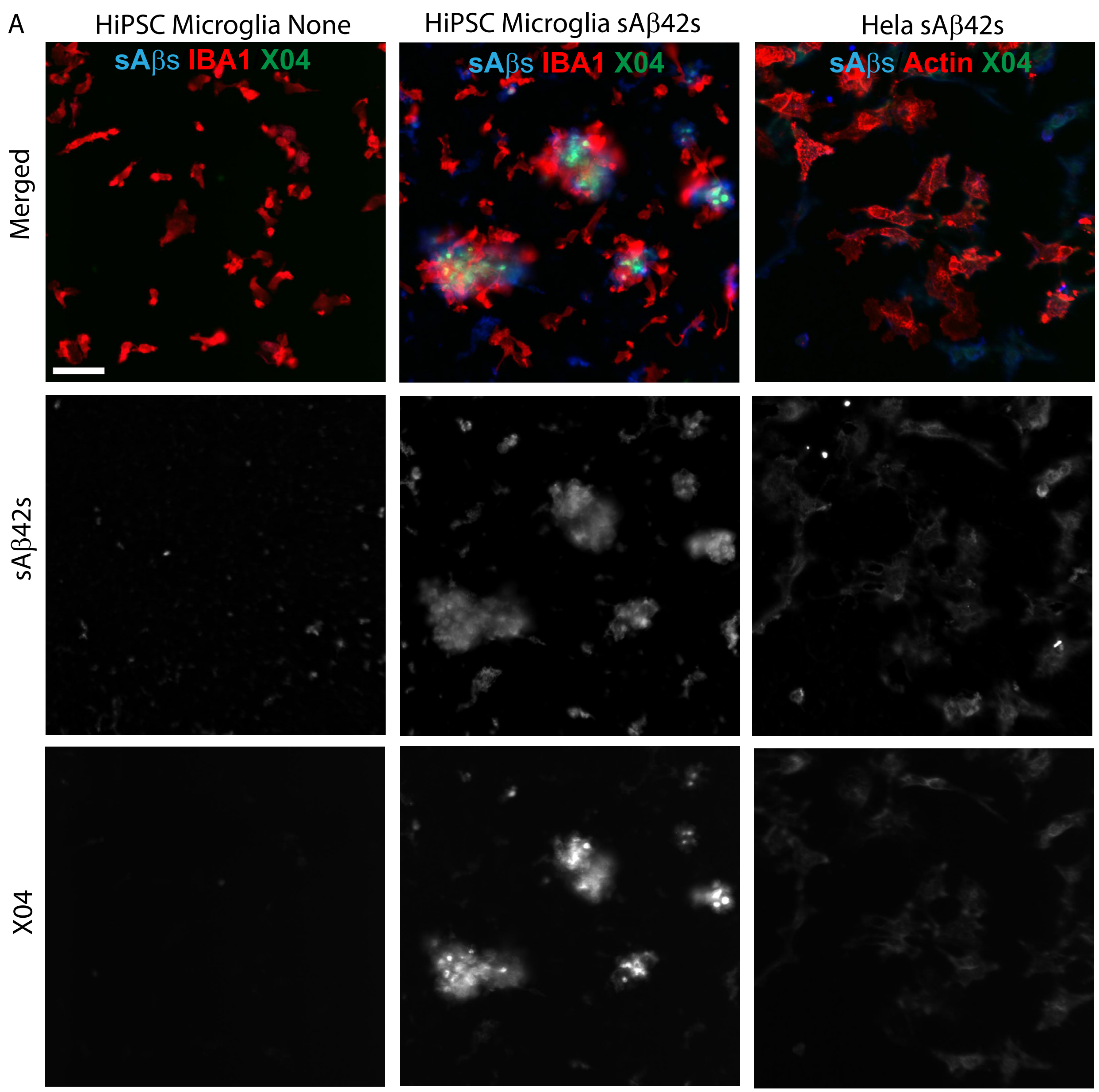

Supplementary Fig. 6 | Amyloid plaque-like structures generated by human iPSC microglia but not by HeLa cells. (A) Left, human iPSC-derived microglia (IBA1, red) receiving no treatment show no accumulation of Aβ (6E10, blue), no plaque-like structures (Methoxy-X04, green). Middle, human iPSC-derived microglia (IBA1, red) treated with 2.5μM sAβ42s (6E10, blue) show accumulation of discrete plaque-like structures (Methoxy-X04, green) that are surrounded by cells. Right, HeLa cells (Phalloidin, red) treated with 2.5μM sAβ42s (6E10, blue) showed low surface binding of Aβ but did not generate the same characteristic plaque-structures (Methoxy-X04, green) observed in human iPSC derived-microglia. All scale bars = 50μm.

Supplementary Fig. 7

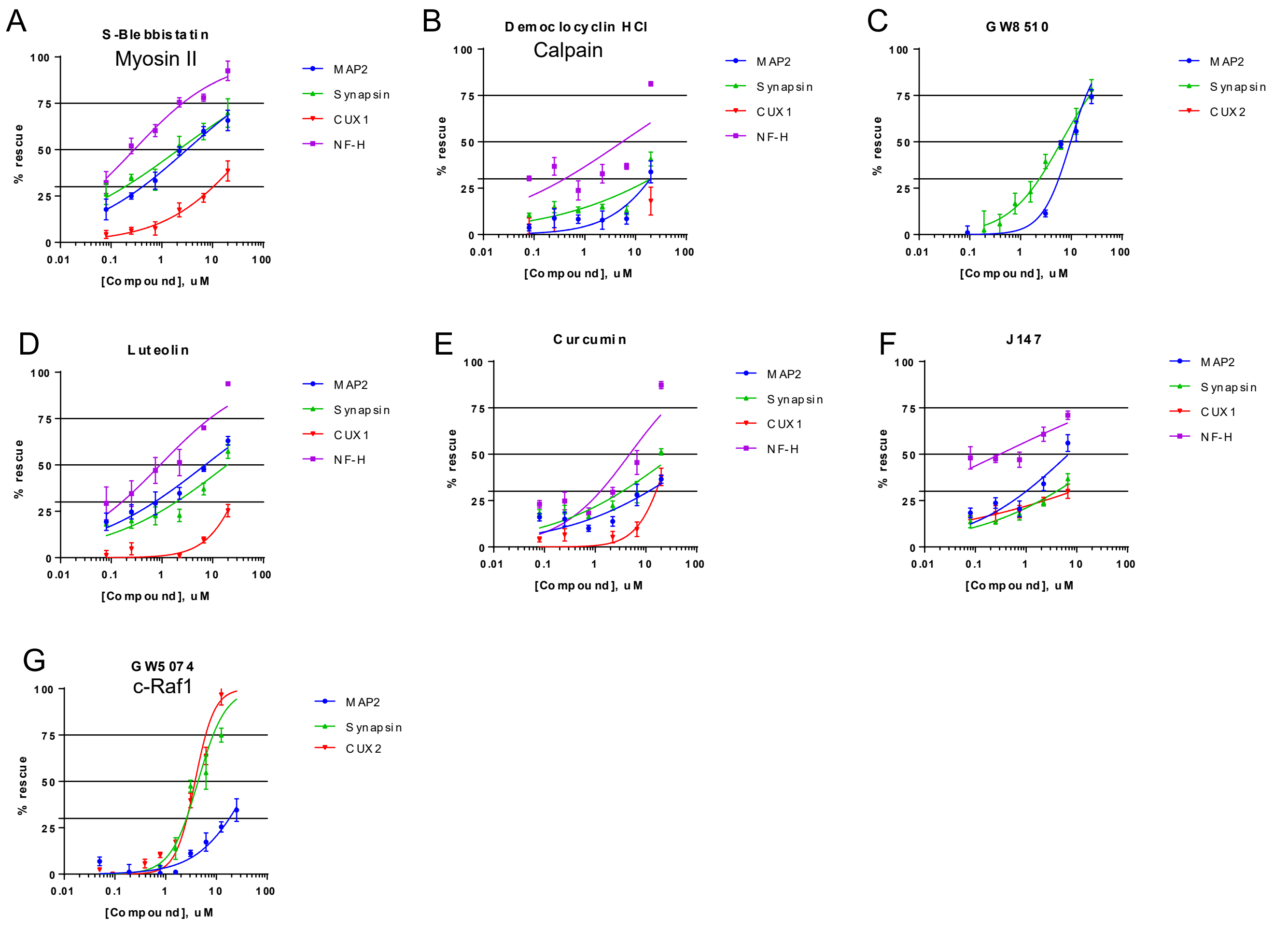

Supplementary Fig. 7 | IC50 validation of focused screen hits. (A-G) Hits from the focused screen (Fig. 5) were tested in dose-response curve for markers MAP2 (blue), Synapsin (green), CUX1/2 (red), NF-H (purple). All error bars represent s.e.m. and n=4 wells. IC50 curves were fitted using Prism software.

Supplementary Fig. 8

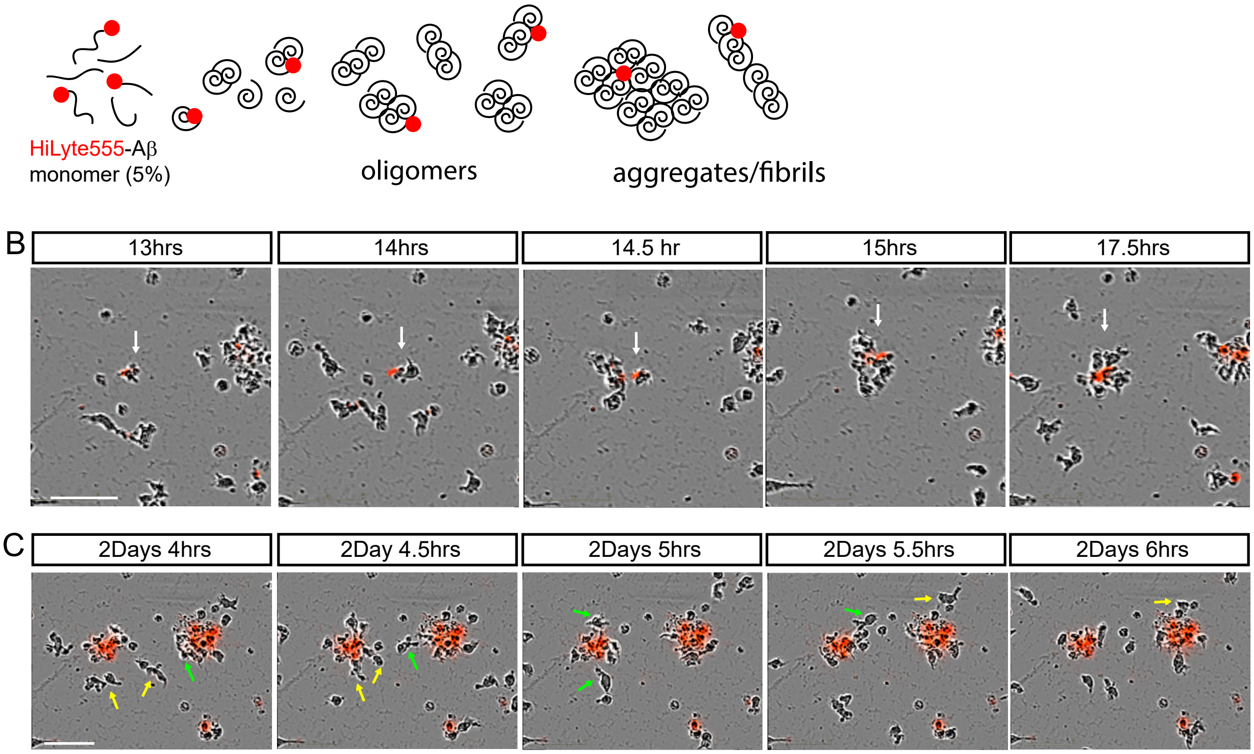

Supplementary Fig. 8 | Human iPSC microglia exhibit dynamic activities during Aβ plaque formation. (A) Schematic showing sAβ42s that were made using 5% HiLyte-555 labeled Aβ42 monomers. (B) Representative images are taken from Incucyte Zoom software over a 7-day time-lapse showing the same field of view to track the microglial formation of one Aβ42 plaque (red) indicated by the white arrow in the indicated time frame. Scale bar = 50 µm. (C) Example of microglia movement around the plaques. After 2 days plaque formation has occurred within this 2-hour window, some microglial cells join plaque indicated by yellow arrows, and some cells that leave plaque indicated by green arrows. Scale bar = 50 µm.

Supplementary Fig. 9

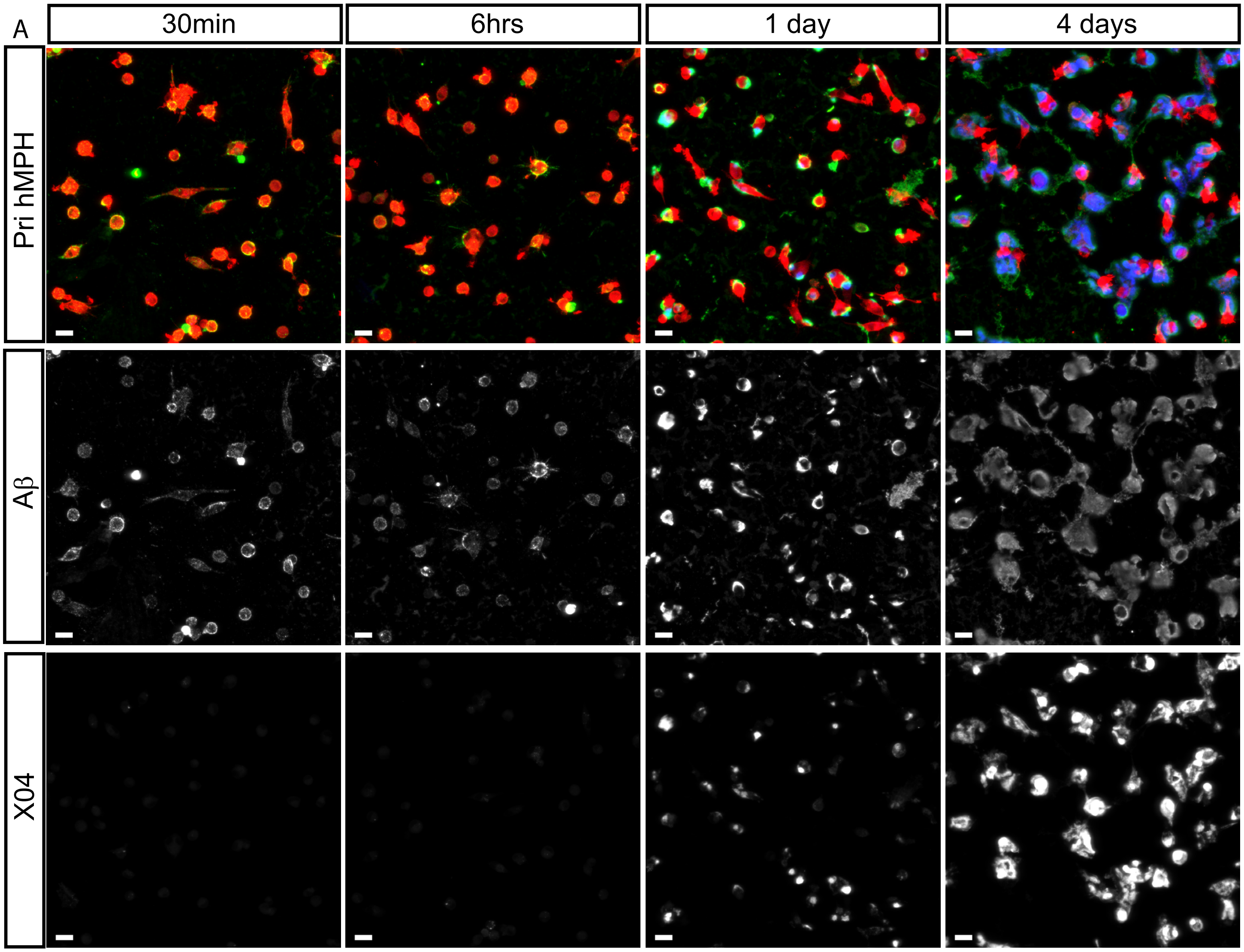

Supplementary Fig. 9 | Human primary macrophages generate internalized Aβ-plaque. (A) Human CD14-derived macrophages were treated with 5 μM sAβ42s, then fixed and stained after 30 minutes, 6 hours, 1 day, and 4 days. Macrophages (IBA1, red) continuously internalize Aβ (green; white - second row) over the course of 4 days and form intracellular X04-positive (blue; white - bottom row) aggregates. All scale bars = 50μm.

Supplementary Fig. 10

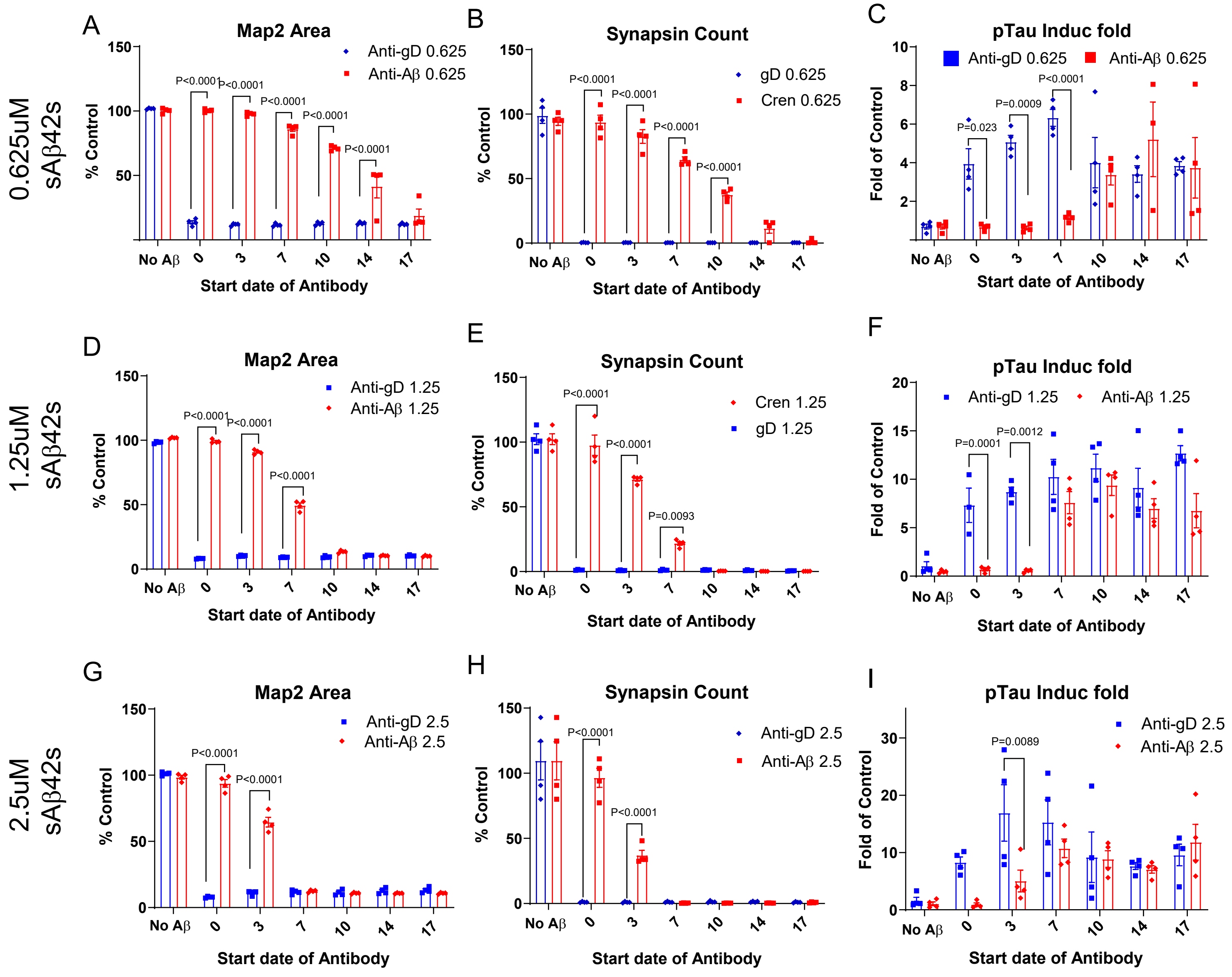

Supplementary Fig. 10 | Anti-Aβ antibody intervention window reduced by faster disease progression. (A-C) Repeated dosing schedule of 12-week old human iPSC neuron cultured with twice a week dosed 0.625 μM of sAβ42s. 0.625 μM Anti-Aβ antibodies (red) or anti-gD control antibodies (blue) were started at indicated time points for repeated dosing regimens. All cells were treated in the same plate and fixed at 21 days post-first dose. MAP2 area (A), synapsin count (B), and p-Tau induction fold (C) were quantified. (D-F) Same experimental set-up but with 1.25 μM of sAβ42s, 1.25 μM Anti-Aβ antibodies (red) or anti-gD control antibodies (blue). (G-I) Same experimental setup but with 2.5 μM of sAβ42s, 2.5 μM Anti-Aβ antibodies (red) or anti-gD control antibodies (blue). Data are presented as mean values +/- SEM and n=4 wells. Two-way ANOVA with Tukey's multiple comparisons test.

Supplementary Movie 1 | Human iPSC microglia exhibit dynamic activities during Aβ plaque formation. Live cell imaging movie (30 minutes/second) using 10X objective and Incucyte Zoom software over a 7-day timelapse shows human iPSC microglia (phase) exhibit dynamic activities and aggregate 5uM 5% HiLyte-555 labeled sAβ42s (red) into Aβ plaque-like structures.

Supplementary Movie 2 | Human CD14-derived macrophages continuously internalize Aβ and do not form extracellular plaques. Live cell imaging movie (30 minutes/second) using 10X objective and Incucyte Zoom software over a 7-day timelapse shows human CD14-derived macrophages (phase) show low motility when treated with 5uM 5% HiLyte-555 labeled sAβ42s (red) and continuously internalize Aβ

Supplementary Movie 3 | Human iPSC microglia exhibit dynamic activities during Aβ plaque formation. Live cell imaging movie (30 minutes/second) using 10X objective and Incucyte Zoom software over a 7-day timelapse with human iPSC microglia (phase) treated with 5uM sAβ42s labeled by HiLyte555 and pHrodo Green. Microglia (phase) continuously internalize Aβ (green) before plaque formation (red) in the center of a group of cultured microglia.

Supplementary Table 1. Description of small molecules used in the focused screen.

Name	Cat. No.	Vendor	Bioactivity Information/ Description	Dose	Neuroprotective citation
PD0325901	4192	Tocris	MEK1/2 inhibitor; Prevents neuronal death caused by oxidative stress	5 mg/kg	Ku et al. 2018
LM22A4	4607	Tocris	TrkB agonist; Neurotrophic	0.001-1000 nM	Massa et al. 2010
7,8-Dihydroxyflavone	3826	Tocris	TrkB agonist. Neurotrophic	5 mg/kg	Andero et al. 2012
LM11A 31 dihydrochloride	5046	Tocris	p75NTR agonist; Increase survival signaling and inhibit amyloid-β-induced degenerative signaling	50 mg/kg	Simmons et al. 2014
(S)-(-)-Blebbistatin	1852	Tocris	Myosin II ATPase inhibitor; Prevents oxidative stressinduced neuronal apoptosis	1 µM	Wang et al. 2017
BI-6C9	Sc-210915A	Santa Cruz Biotech	tBID inhibitor; Protects from glutamate-induced neuronal death	10 µM	Landshamer et al. 2008
Bongkrekic acid solution	B6179	Sigma	ANT inhibitor; Protects against NMDA receptor-mediated neuronal apoptosis	4-16 µg/kg	Muranyi et al. 2005
Sodium butyrate	3850	Tocris	HDAC inhibitor; Anti-inflammatory and neuroprotective	1.2 g/kg	Kilgore et al. 2010
Trichostatin A	1406	Tocris	HDAC inhibitor; Anti-inflammatory and neuroprotective	5-10 mg/kg	Fleiss et al. 2012
Calpeptin	sc-202516	Santa Cruz Biotech	Calpain-2 inhibitor; Prevents neuronal apoptosis	2 µM	Das et al. 2006
Kynurenic Acid Sodium Salt	3694	Tocris	Nonspecific antagonist of excitatory amino acid receptors; Protects from glutamate-induced neuronal death	300 mg/kg	Leib et al. 1996
Necrostatin-1	sc-200142	Santa Cruz Biotech	RIPK1 inhibitor; Block necroptosis and protect dopaminergic neurons	0.1-100 µM	Degterev et al. 2005
BAX Inhibiting Peptide V5	B1436	Sigma	BAX inhibitor; Inhibits neuronal apoptosis	5 µL, 5 mg/mL	Wang et al. 2010
Ivachtin	2788-5	BioVision	Caspase-3 inhibitor; Inhibits neuronal apoptosis	0.5-50 µM	Poksay et al. 2017
Cdk2 Inhibitor II	219445	Calbiochem	CDK2 inhibitor; Inhibits neuronal apoptosis triggered	4 µM	Ye et al. 2010

			by inappropriate activation of CDK		
SB 218078	2560	Tocris	Chk1 inhibitor	5 µM	Gonzalez et al. 2015
PD 0332991 isethionate	4786	Tocris	CDK inhibitor; Inhibits neuronal apoptosis triggered by inappropriate activation of CDK	100 mg/kg	Marathe et al. 2015
Purvalanol A	1580	Tocris	CDK inhibitor; Inhibits neuronal apoptosis triggered by inappropriate activation of CDK	75 nM	Kuruva et al. 2016
Olomoucine	1284	Tocris	CDK inhibitor; Inhibits neuronal apoptosis triggered by inappropriate activation of CDK	1-100 µM	Di Giovanni et al. 2005
GW8510	G7791	Sigma	CDK2 inhibitor; Inhibits neuronal apoptosis triggered by<br>Inappropriate activation of CDK	1-10 µM	Johnson et al. 2005
SB216763	S1075	Selleckchem	GSK-3β Inhibitor; Protects against axon degeneration	3 µM	Liang and Chuang 2006
TDZD-8	ALX-270-354-M005	Enzo	GSK-3β Inhibitor; Protects against axon degeneration	3.3 & 10 µM	Martinez et al. 2002
IM-12	SML0084	Sigma	GSK-3β Inhibitor; Protects against axon degeneration	1 µM	Shan et al. 2017
CHIR 99021 trihydrochloride	4953	Tocris	GSK-3β Inhibitor; Protects against axon degeneration	3.1-25 mg/kg	Pan et al. 2011
Saracatinib (AZD0530)	S1006	Selleckchem	Fyn inhibitor; Neuroprotective	2-1000 nM	Nygaard, Dyck and Strittmatter 2014
SU6656	S7774	Selleckchem	Fyn inhibitor; Neuroprotective	5 µM	Johnson et al. 2005
sun11602	4826	Tocris	Fyn inhibitor; Neuroprotective	1 & 3 µM	Murayama et al. 2013
GM 6001	2983	Tocris	Matrix metalloproteinase inhibitor	5 µg/mouse	Shichi et al. 2011
Indirubin-3'-monoxime	1813	Tocris	GSK3β and CDK inhibitor; Protects against axon degeneration; Anti-apoptotic and neuroprotective	0.04-20 µM	Rudhard et al. 2015
AS601245	ALX-270-443-M005	Tocris	JNK inhibitor.; Anti-inflammatory And neuroprotective	0.04-20 µM	Rudhard et al. 2015
P7C3	4076	Tocris	NAMPT activator; Proneurogenic and neuroprotective	5-40 mg/kg	Pieper et al. 2010

Daunorubicin hydrochloride	1467	Tocris	Increases gangliosides (Especially GQ1b) expression in differentiating neuronal cells	0.04-20 µM	Rudhard et al. 2015
MG-132	1748	Tocris	Calpain inhibitor; protease inhibitor	0.04-20 µM	Rudhard et al. 2015
Capsazepine	0464	Tocris	Vanilloid receptor antagonist; Anti-inflammatory	0.04-20 µM	Rudhard et al. 2015
SU 11248	3768	Tocris	Inhibitor of multiple receptor transduction kinases	0.04-20 µM	Rudhard et al. 2015
SU 6668	3335	Tocris	PDGFR, VEGFR and FGFR inhibitor	0.04-20 µM	Rudhard et al. 2015
Ac-Leu-Leu-Nle-CHO	BML-P120-0005	Tocris	calpain I, calpain II, cathepsin L inhibitor; Prevents neuronal apoptosis	0.04-20 µM	Rudhard et al. 2015
MDL 28170	1146	Tocris	Calpain and Cathepsin B inhibitor; Prevents neuronal apoptosis	0.04-20 µM	Rudhard et al. 2015
SB 239063	1962	Tocris	p38 MAPK inhibitor; Protects against axon degeneration	0.04-20 µM	Rudhard et al. 2015
BAY 11-7082	1744	Tocris	NF-κB inhibitor; Anti-inflammatory And neuroprotective	0.04-20 µM	Rudhard et al. 2015
Luteolin	2874	Tocris	Anti-inflammatory, antioxidant and free radical scavenger.<br>Induces Nrf2 and inhibits caspase-3 activation	0.04-20 µM	Rudhard et al. 2015
Teniposide	SML0609	Sigma	Topoisomerase II inhibitor; Inhibits DNA synthesis	0.04-20 µM	Rudhard et al. 2015
2-TEDC	0645	Tocris	5-, 12-, -15- lipoxygenase inhibitor; Protects against axon degeneration	0.04-20 µM	Rudhard et al. 2015
SB 415286	1617	Tocris	GSK-3β inhibitor; Protects against axon degeneration	0.04-20 µM	Rudhard et al. 2015
FK 506	3631	Tocris	Calcineurin inhibitor; Neuroprotective	0.5-1 mg/kg	Sierra-Paredes and Sierra-Marcuno
STEARDA	2204	Tocris	5-LO (5-lipoxygenase) inhibitor; Protects against axon degeneration	0.04-20 µM	Rudhard et al. 2015
Arctigenin	1777	Tocris	MKK1and IKBa inhibitor, neuroprotective by binding to kainate receptors	0.04-20 µM	Rudhard et al. 2015
Lycorine hydrochloride	HY-N0289	MedChemEx p ress	p21CIP1/WAF1 activator; Inhibits caspase-3 and prevents apoptosis	0.04-20 µM	Rudhard et al. 2015
NKH 477	1603	Tocris	Adenylyl cyclase activator	0.04-20 µM	Rudhard et al. 2015

Demeclocycline hydrochloride	HY-17560	MedChemEx p ress	Calpain inhibitor; Prevents neuronal apoptosis	0.04-20 µM	Rudhard et al. 2015
PDI Inhibitor 16F16	SML0021	Sigma	PDI Inhibitor. Prevents apoptosis induced by misfolded proteins	0.5-100 µM	Hoffstrom et al. 2010
JWH 015	1341	Tocris	cannabinoid (CB2) receptor agonist	0.04-20 µM	Rudhard et al. 2015
GW 5074	1381	Tocris	cRaf1 kinase inhibitor	0.04-20 µM	Rudhard et al. 2015
GBR 12783 dihydrochloride	0513	Tocris	Dopamine uptake inhibitor	0.04-20 µM	Rudhard et al. 2015
Baicalein	1761	Tocris	5-, and 12-lipoxygenase Inhibitor; Protects against axon degeneration	0.04-20 µM	Rudhard et al. 2015
GNE-3511	HY-12947	MedChemEx p ress	DLK inhibitor; Protects against neuronal and synaptic loss	0.04-20 µM	Pichon et al
Edaravone	0786	Tocris	Free radical scavenger; Protects from ROS-induced neurotoxicity	1 & 3 mg/kg	Kawasaki et al. 2007
C 646	4200	Tocris	p300/CBP (HAT) inhibitor	20 µM	Formisano et al. 2015
Zileuton	3308	Tocris	5-lipoxygenase (5-LOX) inhibitor	0.04-20 µM	Rudhard et al. 2015
TRO 19622	2906	Tocris	Mitochondrial permeability transition pore inhibitor; Neuroprotective	0.1-10 µM	Bordet et al. 2007
Resveratrol	1418	Tocris	Cyclooxygenase inhibitor. Antioxidant, neuroprotective against Aβ-related neurotoxicity	0.1-50 µM	Bastianetto, Menard, and Quirion
IU1	4088	Tocris	Deubiquitinating enzyme USP14 inhibitor; Reduce protein aggregates and protects from neuronal loss	400 µg/kg	Min et al. 2017
ISR Inhibitor, ISRIB	509584	Calbiochem	Integrated stress response (ISR) Inhibitor; Prevents neuronal cell death through inhibition of amyloid β-induced ATF4 induction	0.5-100 nM	Hosoi et al. 2016
CTPB	ALX-420-033-M005	Enzo Life Sciences	p300 histone acetyltransferase (HAT) activator	0.5-200 µM	Hegarty et al. 2016
Fluorobexarotene	4064	Tocris	RXR agonist; Stimulates the metabolic clearance of Aβ	20 mg/kg	Bachmeier et al. 2013

AK 7	4754	Tocris	SIRT2 inhibitor; Neuroprotective in Huntington's and Parkinson's murine models	10, 20, & 30 mg/kg	Chopra et al. 2012
Epicatechin	HY-N0001	MedChemExp ress	Anti-oxidant and anti-inflammatory; Neuroprotective	10 mg/kg	Pinto et al 2015
Guggulsterone	2013	Tocris	Steroid receptors antagonist; Anti-inflammatory in microglia	30 & 60 mg/kg	Chen, Huang, Ding 2016
Clusterin Protein	2937-HS	R&D systems	Prevents Aβ aggregation and Fibrillization; Anti-apoptotic		Pucci et al. 2008
Neuropathiazol	5186	Tocris	Neuronal differentiation inducer in hippocampal neural progenitors	0.6-5 µM	Wurdak et al. 2010

Supplementary Table 2. Double culture focused screen results

Compound	Known Axon Protection Effect?	Conc. (µM)	% MAP2 rescue	% Cux2 rescue	% Synapse rescue	% B3T rescue
(S)-(-)-Blebbistatin	No	50	34%	23%	12%	49%
		25	34%	5%	13%	38%
		12.5	25%	3%	15%	34%
		6.25	21%	10%	19%	41%
2-TEDC	Yes	50	22%	16%	10%	31%
7,8-Dihydroxyflavone	No	50	6%	42%	-2%	-14%
Ac-Leu-Leu-Nle-CHO	Yes	50	23%	78%	2%	0%
		25	20%	87%	-5%	-11%
		12.5	18%	87%	-4%	0%
		6.25	31%	85%	0%	14%
Calpeptin	Yes	50	36%	96%	15%	20%
		25	39%	91%	9%	7%
		12.5	27%	79%	11%	4%
		6.25	22%	70%	6%	9%
Demeclocycline hydrochloride	Yes	50	39%	33%	27%	63%
		25	23%	6%	15%	35%
MDL 28170	Yes	50	26%	57%	23%	47%
		25	36%	84%	11%	25%
		12.5	33%	74%	9%	22%

		6.25	33%	81%	10%	18%
AK 7	No	50	-17%	0%	-5%	42%
AS601245	Yes	50	64%	123%	19%	-16%
		25	73%	32%	36%	11%
		12.5	67%	13%	57%	20%
		6.25	49%	9%	37%	14%
BAY 11-7082	Yes	25	17%	82%	-13%	14%
		12.5	36%	85%	-3%	31%
		6.25	34%	70%	-2%	38%
Luteolin	Yes	50	55%	19%	37%	66%
		25	26%	12%	13%	29%
MG-132	Yes	50	25%	90%	4%	57%
		25	26%	91%	8%	30%
		12.5	24%	87%	4%	7%
		6.25	18%	80%	-6%	-3%
C 646	No	50	54%	25%	19%	49%
Daunorubicin hydrochloride	Yes	50	-124%	-39%	-27%	101%
		25	-122%	-23%	-27%	73%
GNE-3511	Yes	50	87%	81%	47%	58%
		25	49%	36%	14%	17%
		6.25	32%	18%	7%	15%
GW 5074	Yes	50	54%	162%	8%	48%
		25	19%	115%	8%	14%
		12.5	12%	76%	6%	9%
		6.25	17%	95%	8%	16%
GW8510	No	50	52%	-22%	49%	47%
		25	37%	-18%	38%	31%
		12.5	37%	16%	38%	20%
		6.25	48%	-1%	52%	32%
PD 0332991 isethionate	No	50	-97%	-37%	-15%	69%
Necrostatin-1	Yes	50	37%	10%	15%	11%
NKH 477	Yes	50	44%	45%	-5%	38%
		25	43%	21%	-4%	35%
		12.5	45%	24%	5%	38%
		6.25	39%	21%	7%	38%
PDI Inhibitor 16F16	No	50	-12%	34%	-13%	-22%
		25	-10%	66%	-8%	-46%
		12.5	1%	45%	-1%	3%
		6.25	6%	46%	5%	27%

Saracatinib (AZD0530)	No	25	65%	18%	10%	30%
		12.5	71%	13%	34%	56%
		6.25	61%	7%	32%	62%
SU6656	No	50	3%	106%	11%	-188%
		25	3%	126%	15%	-37%
		12.5	4%	120%	11%	3%
		6.25	5%	101%	13%	4%
SB 218078	No	50	-92%	-39%	-15%	72%
SB 415286	Yes	50	19%	-13%	12%	39%
SU 11248	Yes	25	16%	59%	4%	-19%
		50	7%	115%	21%	4%
Teniposide	Yes	50	21%	-18%	6%	34%

Supplementary Table 3. Triple culture focused screen results

Compound	Known Axon Protection Effect?	Conc. (µM)	% MAP2 rescue	% Synapse rescue	% B3T rescue
TRO 19622	No	12.50	-36	-13%	33%
		3.13	-25%	-10%	34%
AK 7	No	50.00	28%	17%	56%
		12.50	53%	42%	81%
		3.13	18%	13%	77%
		0.78	1%	3%	79%
Resveratrol	No	50.00	84%	78%	78%
		12.50	57%	49%	54%
		3.13	33%	27%	30%
		0.78	33%	24%	27%
Epicatechin	No	50.00	28%	20%	30%
IU1	No	12.50	32%	23%	41%
Guggulsterone	No	50.00	19%	17%	36%
ISR Inhibitor, ISRIB	No	50.00	31%	21%	22%
C 646	No	50.00	37%	39%	53%
		12.50	41%	38%	42%
		0.78	37%	28%	27%
Neuropathiazol	No	50.00	27%	28%	35%

		12.50	44%	42%	35%
Fluorobexarotene	No	12.50	40%	33%	33%
		3.13	43%	34%	50%
GNE-3511	Yes	50.00	66%	56%	65%
		12.50	102%	81%	82%
		3.13	95%	77%	81%
		0.78	91%	79%	85%
Ac-Leu-Leu-Nle-CHO	Yes	12.50	48%	18%	44%
		3.13	55%	38%	57%
		0.78	21%	14%	38%
2-TEDC	Yes	50.00	48%	40%	73%
		12.50	25%	17%	43%
		3.13	22%	15%	40%
NKH 477	Yes	50.00	53%	38%	81%
		12.50	9%	8%	49%
		3.13	17%	12%	42%
MDL 28170	Yes	50.00	47%	41%	73%
		12.50	31%	8%	33%
		0.78	19%	10%	30%
SB 415286	Yes	50.00	42%	40%	78%
FK 506	No	12.50	34%	34%	52%
PDI Inhibitor 16F16	No	50.00	-5%	-1%	42%
		12.50	-6%	8%	43%
		3.13	44%	43%	71%
BAY 11-7082	Yes	50.00	21%	-1%	44%
STEARDA	No	12.50	24%	21%	33%
		3.13	28%	24%	33%
		0.78	30%	24%	27%
JWH 015	No	50.00	38%	26%	42%
		12.50	50%	43%	60%
Luteolin	Yes	50.00	73%	75%	91%
Teniposide	Yes	50.00	-19%	3%	52%
		12.50	-17%	3%	50%
		3.13	-10%	5%	42%
		0.78	-7%	1%	35%
Lycorine hydrochloride	Yes	50.00	88%	75%	79%
		12.50	93%	77%	83%
		3.13	99%	80%	77%
		0.78	57%	43%	37%
Olomoucine	No	50.00	-8%	-15%	69%

Saracatinib (AZD0530)	No	12.50	-12%	-2%	35%
		3.13	73%	65%	99%
		0.78	72%	65%	99%
P7C3	No	50.00	-8%	2%	43%
		3.13	2%	3%	30%
GW8510	No	50.00	-8%	-15%	63%
		12.50	28%	32%	68%
		3.13	15%	25%	70%
		0.78	-4%	8%	45%
SU6656	No	50.00	40%	36%	48%
		12.50	53%	49%	71%
		3.13	72%	64%	92%
		0.78	58%	51%	80%
Daunorubicin hydrochloride	Yes	0.78	74%	52%	68%
SB216763	No	50.00	-24%	-15%	46%
sun11602	No	50.00	14%	11%	47%
		12.50	19%	15%	43%
		3.13	10%	11%	33%
MG-132	Yes	50.00	25%	-11%	46%
		12.50	18%	-7%	35%
TDZD-8	No	50.00	-29%	-8%	54%
Capsazepine	Yes	12.50	44%	44%	50%
Indirubin-3'-monoxime	Yes	50.00	23%	34%	49%
		12.50	37%	44%	74%
		3.13	6%	9%	31%
SU 11248	Yes	3.13	88%	77%	74%
		0.78	73%	68%	69%
CHIR 99021 trihydrochloride	No	50.00	58%	46%	66%
AS601245	Yes	50.00	95%	45%	44%
		12.50	74%	26%	26%
		3.13	54%	17%	18%
SU 6668	No	50.00	80%	75%	77%
		3.13	31%	26%	34%
Calpeptin	Yes	50.00	34%	34%	66%
SB 218078	No	50.00	53%	19%	-11%
		0.78	10%	14%	38%
BI-6C9	No	50.00	58%	55%	65%
PD0325901	No	50.00	27%	27%	59%
		12.50	8%	8%	43%
		3.13	23%	21%	52%

		0.78	19%	15%	48%
BAX Inhibiting Peptide V5	No	12.50	-6%	14%	32%
Trichostatin A	No	50.00	-13%	1%	41%
		12.50	-18%	2%	31%
(S)-(-)-Blebbistatin	No	50.00	24%	20%	36%
PD 0332991 isethionate	No	50.00	-79%	-29%	90%
Purvalanol A	No	3.13	15%	24%	33%
