# Supplementary material for: Improved modeling of human AD with an automated culturing platform for iPSC neurons, astrocytes and microglia
Source: Nat Commun. 2021 Sep 1;12:5220. doi: 10.1038/s41467-021-25344-6 (PMC8410795; doi:10.1038/s41467-021-25344-6)
Supplement: Supplementary file 2 — Description of Additional Supplementary Files [file 41467_2021_25344_MOESM2_ESM.pdf]

Description of Additional Supplementary Files

Supplementary Movie 1 | Human iPSC microglia exhibit dynamic activities during Aβ plaque formation. Live cell imaging movie (30 minutes/second) using 10X objective and Incucyte Zoom software over a 7-day timelapse shows human iPSC microglia (phase) exhibit dynamic activities and aggregate 5uM 5% HiLyte-555 labeled Aβ42 monomers (red) into Aβ plaque-like structures.

Supplementary Movie 2 | Human CD14-derived macrophages continuously internalize Aβ and do not form extracellular plaques. Live cell imaging movie (30 minutes/second) using 10X objective and Incucyte Zoom software over a 7-day timelapse shows human CD14-derived macrophages (phase) show low motility when treated with 5uM 5% HiLyte-555 labeled Aβ42 monomers (red) and continuously internalize Aβ

Supplementary Movie 3 | Human iPSC microglia exhibit dynamic activities during Aβ plaque formation. Live cell imaging movie (30 minutes/second) using 10X objective and Incucyte Zoom software over a 7-day timelapse with human iPSC microglia (phase) treated with 5uM soluble Aβ species labeled by HiLyte555 and pHrodo Green. Microglia (phase) continuously internalize Aβ (green) before plaque formation (red) in the center of a group of cultured microglia.
