# Supplementary material for: Improved modeling of human AD with an automated culturing platform for iPSC neurons, astrocytes and microglia
Source: Nat Commun. 2021 Sep 1;12:5220. doi: 10.1038/s41467-021-25344-6 (PMC8410795; doi:10.1038/s41467-021-25344-6)
Supplement: Supplementary file 6 — Reporting summary [file 41467_2021_25344_MOESM6_ESM.pdf]

nature research

Corresponding author(s): Ben Chih

Last updated by author(s): Jun 21, 2021

Reporting Summary

Nature Research wishes to improve the reproducibility of the work that we publish. This form provides structure for consistency and transparency in reporting. For further information on Nature Research policies, see Authors & Referees and the Editorial Policy Checklist .

Statistics

For all statistical analyses, confirm that the following items are present in the figure legend, table legend, main text, or Methods section.

n/a	Confirmed	
☐	☒	The exact sample size (*n*) for each experimental group/condition, given as a discrete number and unit of measurement
☐	☒	A statement on whether measurements were taken from distinct samples or whether the same sample was measured repeatedly
☐	☒	The statistical test(s) used AND whether they are one- or two-sided<br>*Only common tests should be described solely by name; describe more complex techniques in the Methods section.*
☒	☐	A description of all covariates tested
☐	☒	A description of any assumptions or corrections, such as tests of normality and adjustment for multiple comparisons
☐	☒	A full description of the statistical parameters including central tendency (e.g. means) or other basic estimates (e.g. regression coefficient) AND variation (e.g. standard deviation) or associated estimates of uncertainty (e.g. confidence intervals)
☐	☒	For null hypothesis testing, the test statistic (e.g. *F*, *t*, *r*) with confidence intervals, effect sizes, degrees of freedom and *P* value noted<br>*Give P values as exact values whenever suitable.*
☒	☐	For Bayesian analysis, information on the choice of priors and Markov chain Monte Carlo settings
☒	☐	For hierarchical and complex designs, identification of the appropriate level for tests and full reporting of outcomes
☒	☐	Estimates of effect sizes (e.g. Cohen's *d*, Pearson's *r*), indicating how they were calculated

Our web collection on statistics for biologists contains articles on many of the points above.

Software and code

Policy information about availability of computer code

Data collection	IN Cell Analyzer 6000 software version 7.2 (GE Healthcare Life Sciences) was used to collect multiplex fluorescent images.<br>ImageXpress Micro Confocal Software version 6.5 (Molecular Device) was used to collect multiplex fluorescent microglia images requiring z stacks (10 sections).<br>IncuCyte ZOOM software version 2018A (Sartorius) was used to acquire time lapse movies.<br>Gyros version 6.4 (Gyros Protein Technologies) was used to measure soluble Aβ42 immunoassay.<br>Compass for Simple Western (Protein Simple) was used for Western Blotting.
Data analysis	IN Cell Developer Toolbox analysis software version 1.93 (GE Healthcare Life Sciences) was used to segment and quantify cellular regions of interest such as dendrites, neuclus, axons, synapse, plaque, microglia, on acquired fluorescent images.<br>Compass 5.0 for Simple Western (Protein Simple) was used to quantify band intensities for western blotting.<br>GraphPad Prism version 8 was used for statistical analysis.

For manuscripts utilizing custom algorithms or software that are central to the research but not yet described in published literature, software must be made available to editors/reviewers. We strongly encourage code deposition in a community repository (e.g. GitHub). See the Nature Research guidelines for submitting code & software for further information.

Data

Policy information about availability of data

All manuscripts must include a data availability statement. This statement should provide the following information, where applicable:

- Accession codes, unique identifiers, or web links for publicly available datasets
- A list of figures that have associated raw data
- A description of any restrictions on data availability

Full scans of the gels and blots are available in Source data file. The screen results generated in this study are provided in the Supplementary Information file. All raw data used to make all quantitative graphs is included in the Supplementary Information file. All other relevant data are available from the corresponding author

upon reasonable request. All processed data supporting the findings of this study are available within the article and its Supplementary Information files. A reporting summary for this article is available as a Supplementary Information file.

Field-specific reporting

Please select the one below that is the best fit for your research. If you are not sure, read the appropriate sections before making your selection.

- [x] Life sciences
- [ ] Behavioural & social sciences
- [ ] Ecological, evolutionary & environmental sciences

For a reference copy of the document with all sections, see nature.com/documents/nr-reporting-summary-flat.pdf

Life sciences study design

All studies must disclose on these points even when the disclosure is negative.

Sample size

A minimum of three, but for most experiments are four independent biological replicates were conducted in the study. The choice of four was a balance between reproducibility and manageability. We believe the sample size was sufficient as our experiments were highly reproducible within those replicates.

Data exclusions

In some experiments 1 out 4 samples might be excluded as an outlier in the image data. In our focused screen, we excluded several data points where drugs exhibited toxicity at high concentrations in order to perform IC50 curve fitting shown in Figure 5 and Figure S7. We used preestablished criteria prior to data exclusion such that any compounds that showed a downward slope in any markers at a high concentration were excluded from our analyses.

Replication

All attempts at replication were successful for all experiments that were replicated. The p-c-Jun Western Blot (Figure 5J) was not replicated as this experiment was done on the same samples as the Figure 5H staining experiment to corroborate this experiment. In addition, this Western blot was done on 6-month old neurons, and due to the extended time of culturing required, we did not have the opportunity to replicate this experiment. For the Tau Western blots (Figure S2Z), we replicated this experiment independently 4 times and all replication attempts were successful.

Randomization

Our automated culture system has shown to have a very good well to well consistency and no plate positional effect. No randomization was done on in vitro studies. Allocation was not random due to the complexity of our 384-well plate maps. However, covariates such as edge effect were controlled for as our culture system removes edge effect from our plates. To characterize the variability of the assay performance, average z-factors (a measure of assay reliability) were calculated from multiple batches and experiments (10-20) for the aforementioned assays. They ranged from 0.5–0.7 (Fig. 1Z) indicating robust assay in all well placements. Therefore, we felt that randomized allocation was not relevant for our study.

Blinding

Investigators were not blinded during experimental setup due to the complicated nature of our 384-well plate maps and the large number of conditions tested. We felt that introducing blinding during experimental setup would increase the risk of error during experimental setup. Plates are cared for, fixed, and stained using automation techniques, controlling much of the bias during experimental setup. In addition, imaging techniques are performed in a systematic, automated way such that data acquisition is blinded. In addition, we used an automated image analysis platform to reduce bias during image analysis.

Reporting for specific materials, systems and methods

We require information from authors about some types of materials, experimental systems and methods used in many studies. Here, indicate whether each material, system or method listed is relevant to your study. If you are not sure if a list item applies to your research, read the appropriate section before selecting a response.

Materials & experimental systems

n/a	Involved in the study
[ ]	[x] Antibodies
[ ]	[x] Eukaryotic cell lines
[x]	[ ] Palaeontology
[x]	[ ] Animals and other organisms
[x]	[ ] Human research participants
[x]	[ ] Clinical data

Methods

n/a	Involved in the study
[x]	[ ] ChIP-seq
[x]	[ ] Flow cytometry
[x]	[ ] MRI-based neuroimaging

Antibodies

Antibodies used

All antibodies used in the study are listed in the method.

Antibodies used for microglia marker expression immunostaining included Goat anti TREM2 (1:500, R&D Systems, AF1828), Mouse anti MERTK (1:500, BioLegend, 367602), Rabbit anti IBA1 (1:1000, Wako Chem, 019-19741), Rabbit anti TMEM119 (1:500, Abcam, ab209064), Mouse anti CD33 (1:500, BioLegend, 303302), Rabbit anti CX3CR1 (1:500, BioRad, AHP1589), Mouse anti CD64 (1:500, BioLegend, 305012), Rabbit anti P2RY12 (1:500, Sigma, HPA014518), Mouse anti CD32 (1:500, BioLegend,

303212), Rabbit anti PU.1 (1:500, Cell Signaling, 2266S)

Antibodies used for other immunostaining included Chicken anti MAP2 (1:2000, GeneTex, GTX85455), Guinea pig anti Synapsin ½ (1:750, Synaptic Systems, #106 004), Mouse anti Tau HT7 (1:500, Invitrogen, MN1000), Mouse anti A! 6E10 (1:500, BioLegend, #803003), Rabbit anti CUX2 (1:500, Abcam, ab140329), Rat anti CTIP2 (1:500, Abcam, ab18465), Guinea pig anti vGlut2 (1:1000 , Synaptic systems, 135304), Mouse anti Shank (1:200 , Millipore N23B/49), Mouse anti PSD95 (1:200 ,Millipore, K28/43, MABN68), Mouse anti GluR1 (1:200 , Synaptic Systems, 182011), Mouse anti PanShank (1:200 , Millipore, N23B/49), Mouse anti GluR2 (1:200 , Millipore, 14C12.2), Mouse anti PanSAPAP (1:200 , Millipore, N127/31, MABN54), Mouse anti NR1 (1:200 , Millipore, 54.1), Rabbit anti phospho-Tau S396-404 (1:3000, Genentech), Rabbit anti phospho-Tau S235 (1:1000, Thermofisher, PA5-35761), Mouse anti !- Tubulin Tuj1 (1:500, BioLegend, #801202), Chick anti Neurofilament-Heavy Chain (1:3000, Abcam, ab4680), Rabbit anti Iba1 (1:1000, Wako Chem, 019-19741), Rabbit anti TMEM119 (1:500, Abcam, ab209064), Rabbit anti ApoE (1:500, Thermofisher 16H22L18), Rabbit anti APP (1:500, Abcam ab32136), Guinea pig anti GFAP (1:500 Synaptic System 173004), Rabbti anti ALDH1L1 (1:500, Abcam ab190298), Rabbit anti Vimentin (1:500, Cell signaling 3932S), Rabbit anti EAAT1 (1:500, Boster PA2185).

Antibodies used for Gyros immunoassays and Abeta conformational ELISA assays included Mouse anti A! 6E10 (100ng/ml), BioLegend, #803003), Rabbit beta-Amyloid H31L21 (25nM, Thermofisher, #700254), Mouse beta-Amyloid antibody GT622 (100ng/ml, Genetex, GTX635160), Rabbit anti-amyloid fibrils OC antibody (100ng/ml, Millipore, AB2286).

Secondary antibodies used for immunostaining were from Jackson ImmunoResearch. They were of donkey host, with IgG (H+L) specificity against primary antibody host species listed above. DyLight™ 405 AffiniPure Donkey Anti-Chicken IgY (IgG) (H+L) (703-475-155), Alexa Fluor® 488 AffiniPure Donkey Anti-Chicken IgY (IgG) (H+L) (703-545-155), Cy™3 AffiniPure Donkey Anti-Chicken IgY (IgG) (H+L) (703-165-155), Alexa Fluor® 647 AffiniPure Donkey Anti-Chicken IgY (IgG) (H+L) (703-605-155), DyLight™ 405 AffiniPure Donkey Anti-Guinea Pig IgG (H+L) (706-475-148), Alexa Fluor® 488 AffiniPure Donkey Anti-Guinea Pig IgG (H+L) (706-545-148), Cy™3 AffiniPure Donkey Anti-Guinea Pig IgG (H+L) (706-165-148), Alexa Fluor® 647 AffiniPure Donkey Anti-Guinea Pig IgG (H+L) (706-605-148), DyLight™ 405 AffiniPure Donkey Anti-Mouse IgG (H+L) (715-475-151), Alexa Fluor® 488 AffiniPure Donkey Anti-Mouse IgG (H+L) (715-545-151), Cy™3 AffiniPure Donkey Anti-Mouse IgG (H+L) (715-165-151), Alexa Fluor® 647 AffiniPure Donkey Anti-Mouse IgG (H+L) (715-605-151), DyLight™ 405 AffiniPure Donkey Anti-Rabbit IgG (H+L) (711-475-152), Alexa Fluor® 488 AffiniPure Donkey Anti-Rabbit IgG (H+L) (711-545-152), Cy™3 AffiniPure Donkey Anti-Rabbit IgG (H+L) (711-165-152), Alexa Fluor® 647 AffiniPure Donkey Anti-Rabbit IgG (H+L) (711-605-152), DyLight™ 405 AffiniPure Donkey Anti-Rat IgG (H+L) (712-475-153), Alexa Fluor® 488 AffiniPure Donkey Anti-Rat IgG (H+L) (712-545-153), Cy™3 AffiniPure Donkey Anti-Rat IgG (H+L) (712-165-153), Alexa Fluor® 647 AffiniPure Donkey Anti-Rat IgG (H+L) (712-605-153).

Validation

Validation of all antibodies were taken from manufacturer's websites:
TREM2,R&D Systems,AF1828,Human,"WB, ICC, ELISA. Detects human TREM2 in direct ELISAs and Western blots. In direct ELISAs, less than 50% cross-reactivity with recombinant mouse TREM2b is observed. Immunogen Mouse myeloma cell line NS0-derived recombinant human TREM2 His19-Ser174 Accession # Q9NZC2"
MERTK,BioLegend,367602,Human,"IF, FlowCyt. Immunogen MERTK extracellular domain/Fc fusion. Human monocytes were stimulated and cultured with M-CSF for seven days and stained with CD11b APC and purified human MERTK (clone 590H11G1E3, top)"
IBA1,Wako,019-19741,"Human, rat, mouse","ICC, IHC. Immunogen Synthetic peptide C-terminal of Iba1. "
TMEM119,Abcam,ab209064,Mouse,"IHC-Fr, IHC-P. Knockout validated. Immunogen Recombinant fragment (GST-tag) within Mouse TMEM119 aa 100 to the C-terminus (intracellular)"
CD33,BioLegend,303302,"Human, Chimp",FC and ICC.
CX3CR1,BioRad,AHP1589,"Human, rat, mouse","IHC, WB. Immunogen A peptide corresponding to amino acids 2-21 of human CX3CR1."
CD64,BioLegend,305012,"Human, Baboon, Capuchin Monkey, Chimpanzee, Cynomolgus, Rhesus, Squirrel Monkey","IF, FC. Immunogen Human rheumatoid synovial fluid cells and fibronectin-purified monocytes."
P2RY12,Sigma,HPA014518,Human,"IHC. Enhanced validation by orthogonal RNAseq. Immunogen purinergic receptor P2Y, G-protein coupled, 12."
CD32,BioLegend,303212,"Human, African Green, Baboon, Cynomolgus, Rhesus",IF FC.
PU.1,CellSignaling,2266S,Human,"WB, IP, IHC, IF, FC. Immunogen synthetic peptide N terminus PU1"
MAP2,GeneTex,GTX85455,"Human, Mouse, Rat","WB, ICC/IF, IHC. Chickens were immunized with two synthetic peptide / keyhole limpet hemocyanin (KLH) conjugates. These synthetic peptides corresponded to different regions of the MAP-2 gene product, but are shared between the human (NP_002365, NCBI) and mouse (P20357, NCBI) sequences"
synapsin1/2,Synaptic Systems,106004,"Human, rat, mouse, hamster, cow, zebrafish",Specific for synapsins 1a/b and 2a/b. KO validated. Synthetic peptide corresponding to AA 2 to 28 from rat Synapsin1 (UniProt Id: P09951)
Tau HT7,Invitrogen,MN1000,"Cow, human","WB, IHC, ELISA, IF. Purified human Tau, epitope human Tau between residue 159 and 163 (numbering according to human Tau40), corresponding to the amino acid sequence PPGQK."
abeta 6E10,BioLegend,803003,Human,"WB, ELISA, IHC, ICC (lit). This antibody is reactive to amino acid residue 1-16 of beta amyloid. The epitope lies within amino acids 3-8 of beta amyloid (EFRHDS)."
CUX2,Abcam,ab140329,"Mouse, human predicted","WB. Synthetic peptide, corresponding to a region within N terminal amino acids 126-175 of Mouse Cux2 (NP_031830)"
CTIP2,Abcam,ab18465,"Mouse, human ","ICC, WB, FC. Immunogen Fusion protein corresponding to Human Ctip2. GTS fusion. "
vGlut2,Synaptic Systems,135304,"human, rat, mouse, cow","WB, IP, IHC, ICC, EM, FACS. KO validated. Immunogen Recombinant protein corresponding to AA 456 to 560 from rat VGLUT1"
shank,millipore,N23B/49,"Human, Mouse, Rat","WB, IHC. Immunogen Recombinant protein consisting of SH3/PDZ domain of rat Shank2."
PSD95,millipore,K28/43,"Human, Mouse, Rat","IHC, WB, ICC. Detect PSD95 using this Anti-PSD95 Antibody, clone K28/43 validated for use in IH, WB & IC."

GluR1,Synaptic Systems,182011,"Human, Mouse, Rat","WB, IP, ICC, IHC. KO validated specific to GluA1"
GluR2,millipore,14C12.2,"Human, Mouse, Rat","IHC, WB. Immunogen GST-tagged recombinant rat GluR2 N-terminal extracellular domain fragment."
PanSAPAP,millipore,N127/31,Rat,"IHC, WB. Recombinant protein corresponding to rat SAPAP."
NR1,millipore,54.1,"Human, monkey, rat, xenopus","ELISA, ICC, IHC. Recognizes an epitope between amino acids 660-811 of the NMDAR1 receptor. Shows no cross-reactivity with NMDAR2, NMDAR3, NMDAR4 or NMDAR5."
pTau S235,Thermo,PA5-35761,"Human, Mouse, Rat",WB. Immunogen Synthetic peptide derived from human Tau around the phosphorylation site of Serine 235
betaTubulin Tuj1,BioLegend,801202,"Human, mouse, rat","IHC, ICC, WB, FC (lit). Immunogen This antibody was raised against microtubules derived from rat brain."
NFL H,Abcam,ab4680,"Human, mouse, rat, cow","ICC, IHC, WB. Immunogen native protein purified from cow spinal cord. "
ApoE,Thermo,16H22L18,"Human, mouse, rat","WB, ICC. This Antibody was verified by Relative expression to ensure that the antibody binds to the antigen stated."
APP,abcam,ab32136,"Human, rat, mouse","WB, IHC, IP, ICC. Knockout validated. "
GFAP,Synaptic Systems,173004,"Human, rat, mouse, chicken, sheep. ","WB, IP, ICC, IHC. KO validated specific to GFAP."
ALDH1L1,Abcam,ab190298,"Human, rat.","WB, ICC. Recombinant full length protein corresponding to Human ALDH1L1 aa 1 to the C-terminus. Immunogen expressed and purified from E. coli."
Vimentin,CellSignaling,3932S,"Human, mouse, rat, monkey",WB. Vimentin (R28) Antibody detects endogenous levels of total vimentin protein. KO verified.
EEAT1,Boster,PA2185,"human, mouse, rat","WB. No cross reactivity with other proteins. Immunogen A synthetic peptide corresponding to a sequence at the C-terminus of human EAAT1 (519-537aa MKKPYQLIAQDNETEKPID), different from the related rat and mouse sequences by three amino acids."
phospho-c Jun,Cellsignaling,2361S,"Human, mouse, rat","WB, IHC. Phospho-c-Jun (Ser63) (54B3) Rabbit mAb detects endogenous levels of c-Jun only when phosphorylated at serine 63."
GAPDH,SantaCruz,SC20357,Human,"WB, IHC, ICC. GAPDH (0411) is a mouse monoclonal antibody raised against recombinant GAPDH of human origin. Suitable for use as control antibody for GAPDH"
Histone H3,Cellsignaling,4499S,"Human, mouse, rat, monkey","WB, IHC, ICC, FC. Histone H3 (D1H2) XP® Rabbit mAb detects endogenous levels of total Histone H3 protein, including isoforms H3.1, H3.2, and H3.3. This antibody also detects the Histone H3 variant CENP-A. This antibody does not cross-react with other core histones."
3R Tau,Millipore,05-803,Human,"IHC, WB. Recognizes Tau (3-repeat isoform RD3), Mr 45-65 kDa. Higher MW bands (68-72 kDa) represent phosphorylated Tau."
4R Tau,Millipore,05-804,"Human, rat, cow, monkey","IHC, WB. Recognizes Tau (4-repeat isoform RD4), Mr 45-65 kDa. Higher MW band (68-72 kDa) represents phosphorylated Tau."
GT622,Genetex,GTX635160,Human,"IHC, Dot, ELISA. The immunogen used to generate this antibody corresponds to Beta amyloid (1-42)."
OC fibril,millipore,AB2286,Human,"IP, IHC, ICC, ELISA, WB, DB. This antibody recognizes generic epitopes common to many amyloid fibrils and fibrillar oligomers, but not prefibrillar oligomers or natively folded proteins. It may also show weak reactivity against Aβ monomers while AB2287 does not."

Eukaryotic cell lines

Policy information about cell lines

Cell line source(s)	MTI Global Stem (HIP Neural Stem Cells, BC1 line, GSC-4311)<br>Primary human astrocytes (Thermofisher, N7805100)<br>iCell microglia (Cellular dynamics International, 01279)<br>iPSC microglia (BrainXell Inc)<br>Human M1 Macrophages (GM-CSF) monocyte-derived (promoCell, C-12914)<br>Human M2 Macrophages (M-CSF) monocyte-derived (PromoCell, C-12915)<br>HeLa cells (ATCC, CCL-2)<br>NSC-line2 (Roche, gift from Christoph Patsch)
Authentication	Primary human astrocytes were validated for GFAP expression by ThermoFisher. Furthermore, we validated these cells express cell type specific markers such as GFAP, Vimentin, ALDH1L1, and EAT1.<br>iCell microglia were validated by Cellular Dynamics to exhibit functional characteristics similar to human microglia, including phagocytosis and cytokine-mediated inflammatory responses, and express relevant microglial markers (TREM2, CX3CR1, TMEM119, P2RY12, IBA1). iPSC microglia were validated by BrainXcell where they confirmed expression of microglia surface markers including: CD45, CD11b, CX3CR1, P2RY12, and TMEM119. Immunocytochemistry shortly after plating microglia reveals prominent expression of TREM2 (green) and IBA1 (red). Human iPSC Microglia demonstrate phagocytosis and can be stimulated to secrete a variety of cytokines. All microglial lines were also validated by our lab for cell-type specific markers (Supplementary Figure 5).<br>Human M1 macrophages and Human M2 macrophages were validated by PromoCell as positive for CD80+ and CD68+.<br>BC1 Neural Stem Cells were genotyped in house. Neural stem cells and differentiated neurons were validated using cell type specific markers (Figure 1).<br>NSC-line2 neural stem cells and differentiated neurons were validated using cell type specific markers.<br>HeLa cells were karyotyped and validated by ATCC.

Mycoplasma contamination	All cell lines used for this study were tested negative for mycoplasma contamination
Commonly misidentified lines<br>(See ICLAC register)	No commonly misidentified cell lines were used in this study.
